# Metabolite and Phytohormone Profiling Illustrates Metabolic Reprogramming as an Escape Strategy of Deepwater Rice during Partially Submerged Stress

**DOI:** 10.3390/metabo10020068

**Published:** 2020-02-14

**Authors:** Atsushi Fukushima, Takeshi Kuroha, Keisuke Nagai, Yoko Hattori, Makoto Kobayashi, Tomoko Nishizawa, Mikiko Kojima, Yoshinori Utsumi, Akira Oikawa, Motoaki Seki, Hitoshi Sakakibara, Kazuki Saito, Motoyuki Ashikari, Miyako Kusano

**Affiliations:** 1 RIKEN Center for Sustainable Resource Science, Yokohama, Kanagawa 230-0045, Japan; atsushi.fukushima@riken.jp (A.F.); kobamako@riken.jp (M.K.); tomoko.nishizawa@riken.jp (T.N.); mikiko@riken.jp (M.K.); yoshinori.utsumi@riken.jp (Y.U.); akira.oikawa@riken.jp (A.O.); motoaki.seki@riken.jp (M.S.); sakaki@riken.jp (H.S.); kazuki.saito@riken.jp (K.S.); 2 Bioscience and Biotechnology Center, Nagoya University, Nagoya, Aichi 464-8601, Japan; kurohat236@affrc.go.jp (T.K.); nagai.k@nuagr1.agr.nagoya-u.ac.jp (K.N.); hattori405@gmail.com (Y.H.); ashi@agr.nagoya-u.ac.jp (M.A.); 3 Faculty of Agriculture, Yamagata University, Tsuruoka, Yamagata 997-8555, Japan; 4 Graduate School of Bioagricultural Sciences, Nagoya University, Nagoya, Aichi 464-8601, Japan; 5 Graduate School of Pharmaceutical Sciences, Chiba University, Chuo-ku, Chiba 263-8522, Japan; 6 Graduate School of Life and Environmental Sciences, University of Tsukuba, Tsukuba, Ibaraki 305-8572, Japan; 7 Tsukuba Plant Innovation Research Center, University of Tsukuba, Tsukuba, Ibaraki 305-8572, Japan

**Keywords:** deepwater rice, anoxia, hypoxia, SNORKEL, flooding stress, metabolome, glycolysis

## Abstract

Rice varieties that can survive under submergence conditions respond to flooding either by enhancing internode elongation or by quiescence of shoot elongation. Despite extensive efforts to identify key metabolites triggered by complete submergence of rice possessing *SUBMERGENCE 1* (*SUB1*) locus, metabolic responses of internode elongation of deepwater rice governed by the *SNORKEL 1* and *2* genes remain elusive. This study investigated specific metabolomic responses under partial submergence (PS) to deepwater- (C9285) and non-deepwater rice cultivars (Taichung 65 (T65)). In addition, we examined the response in a near-isogenic line (NIL-12) that has a C9285 genomic fragment on chromosome 12 introgressed into the genetic background of T65. Under short-term submergence (0–24 h), metabolite profiles of C9285, NIL-12, and T65 were compared to extract significantly changed metabolites in deepwater rice under PS conditions. Comprehensive metabolite and phytohormone profiling revealed increases in metabolite levels in the glycolysis pathway in NIL-12 plants. Under long-term submergence (0–288 h), we found decreased amino acid levels. These metabolomic changes were opposite when compared to those in flood-tolerant rice with *SUB1* locus. Auxin conjugate levels related to stress response decreased in NIL-12 lines relative to T65. Our analysis helped clarify the complex metabolic reprogramming in deepwater rice as an escape strategy.

## 1. Introduction

Flooding is an environmental stress that affects plant growth and development. Most irrigated rice (*Oryza sativa*) cultivars are not capable of dealing with flooding. It is known that flood-tolerant rice cultivars have developed two opposing adaptation mechanisms to tolerate flooding: ‘escape strategy’ and ‘quiescence strategy’ [1,2,3]. The flash-flood-tolerant rice plants that adapted a ‘quiescence strategy’ can resume their growth after floodwaters recede [4,5]. In contrast, flood-tolerant rice using an ‘escape strategy’ can tolerate deepwater flooding conditions, such as partial submergence (PS), by rapidly elongating their internodes when water levels rise [2,6]. Many studies on *Arabidopsis* (for example, see [5]), *Oryza* (for example, see [7,8]), *Rumex* [9,10,11,12], *Rorippa* [3,13,14], and *Echinochloa* [15,16] have investigated the molecular mechanisms for withstanding flooding.

Quantitative trait locus (QTL) analysis detected a major QTL known as *SUBMERGENCE 1* (*SUB1*) that controlled submergence tolerance to flash floods in an Indian rice variety, FR13A [17,18]. Subsequent works led to the identification of the gene *SUB1A* as a major contributor to flash-flood tolerance (i.e., complete submergence (CS) in this case) [19,20]. QTL studies of deepwater rice cultivar C9285 revealed that three major QTLs (on chromosomes 1, 3, and 12) regulated rapid internode elongation [21,22,23,24]. In a previous study, we developed near-isogenic line 12 (NIL-12) that possessed a C9285 genomic fragment on chromosome 12 introgressed into a non-deepwater rice cultivar Taichung 65 (T65) genetic background [23]. Hattori and colleagues identified the genes *SNORKEL 1* and *SNORKEL2* (*SK1/2*) on chromosome 12 toward the deepwater response, which is the most effective for the trait [25]. Although the downstream signaling pathways regulated by SK1/2 are largely unknown, phytohormones, such as ethylene, gibberellins, and abscisic acid, are thought to regulate the rapid elongation response [26,27,28].

The use of next-generation sequencing and microarray platforms led to the development of high-throughput transcript profiling (e.g., RNA-Seq) technologies as an efficient functional genomics tool [29,30]. In flash flood-tolerant rice, *Rumex, Rorippa*, and *Echinochloa*, large-scale transcriptome analyses using microarrays or RNA-Seq have been performed to attempt to identify the mechanism controlling flooding tolerance [11,16,31]. Comparative transcriptome analysis with two varieties, C9285 and T65, revealed differential expression patterns between the genotypes [32]. Using a genome-wide association study with a diversity panel of Asian and deepwater rice varieties, along with high-resolution linkage analysis, a recent study identified the gibberellin biosynthesis gene, *SD1* (*SEMIDWARF1*), as a causal gene within the QTL on chromosome 1 for the deepwater response [33].

Recent advances in separation techniques, including gas chromatography (GC), liquid chromatography (LC), and capillary electrophoresis (CE), combined with mass spectrometry (MS), have facilitated the detection of a wide range of primary and secondary metabolites [34,35,36]. To investigate changes in metabolite levels during CS stress, metabolomic approaches using nuclear magnetic resonance (NMR) and/or GC-MS were conducted in rice with the *SUB1A* gene [37,38,39], flood-tolerant and sensitive wheat [40], and soybean [41].

Despite extensive efforts to understand the molecular and biochemical mechanisms associated with CS stress, comprehensive metabolic responses, including phytohormones, during rapid internode elongation remain elusive. Thus, this study investigated the key metabolic responses thought to contribute to internode elongation under PS conditions in deepwater rice C9285 and NIL-12, and non-deepwater rice T65. Using comprehensive analyses that integrated GC-MS, CE-MS, and LC-MS profiling, we uncovered the metabolic impact of the major QTL on chromosome 12, including *SK1/2* [25].

## 2. Results

### 2.1. Effects of Submergence in the Presence or Absence of Light in Deepwater Cultivar C9285

**Figure 1:**
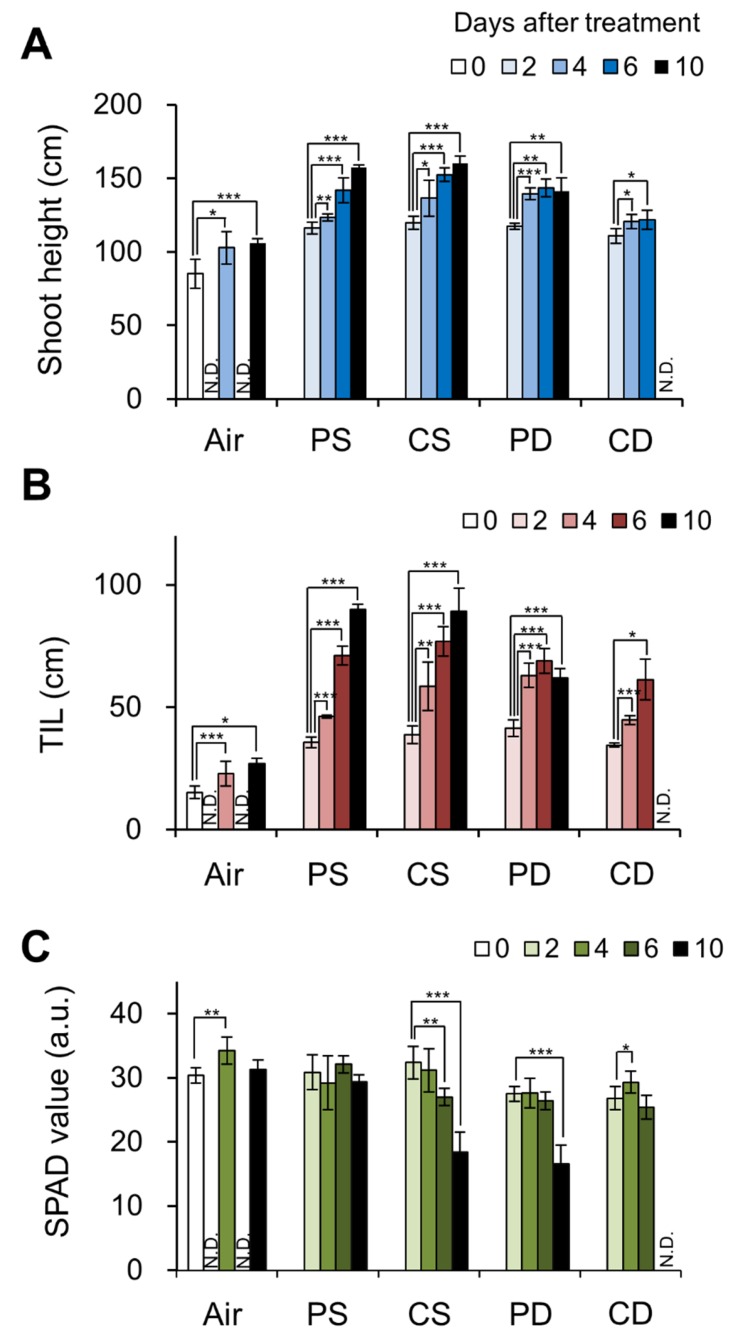
Effects on plant growth and relative amount of chlorophylls of C9285 in submergence and light treatments. Shoot height (**A**), total internode length (TIL) (**B**), and soil plant analysis development (SPAD) value (**C**) of C9285 are shown. We collected each parameter at 2, 4, 6, and 10 days after submergence treatment. We also collected non-submergence treatment data as a control. We were not able to measure shoot height, TIL, and SPAD values at 10 days of CS treatment under dark (CD) because samples were broken when drawn up. Bars represent mean, error bars represent standard deviation of the mean. Air, water level is under the soil surface; PD, PS treatment under dark condition; N.D., Not determined; a.u., arbitrary unit. Number of biological replicates, *n* = 4–10. *t*-test: * *p* < 0.05, ** *p* < 0.005, *** *p* < 0.0005.

To investigate the relationship among rapid internode elongation, energy source, and changes in metabolite levels, we first focused on deepwater rice C9285 under different light and different submergence conditions (Supplementary Figures S1 and S2). We then evaluated growth and amount of chlorophyll in leaf tissue at 2, 4, 6, and 10 days after the four submergence treatments (Figure 1). We also measured the shoot height and the total internode length (TIL). Shoot height and TIL of C9285 increased in a no-submergence condition (Air), as well as in all four treatments (Figure 1A,B). Soil plant analysis development (SPAD) values showed similar trends under Air and PS treatment under light condition conditions, whereas they decreased after four days under CS treatment under light, PS treatment under dark (PD), and CS treatment under dark (CD) (Figure 1C). In particular, after 10 days, C9285 died under CD conditions. These observations suggest that C9285 was healthiest under Air and PS conditions. In addition, C9285 plants showed prominent elongation of shoots and TIL in the four-day-PS condition compared to the Air condition. Therefore, we chose Air and PS conditions for further analysis.

Based on the above results, we designed two types of metabolome experiments: (1) a short-term experiment within 24 h following the PS treatment, and (2) a long-term experiment within 288 h following the PS treatment (Supplementary Figure S3). In the short-term experiment, we used GC-MS to capture changes in primary metabolites that provided energy and building blocks for elongation. To explore the impact of one of the major QTL on chromosome 12, we performed a long-term experiment to compare metabolite changes between NIL-12 and T65 using a multi-platform metabolomics approach (GC, CE, and LC-MS) [42,43].

### 2.2. Short-Term Responses of Metabolite Levels in Deepwater Rice under the PS Condition

**Figure 2:**
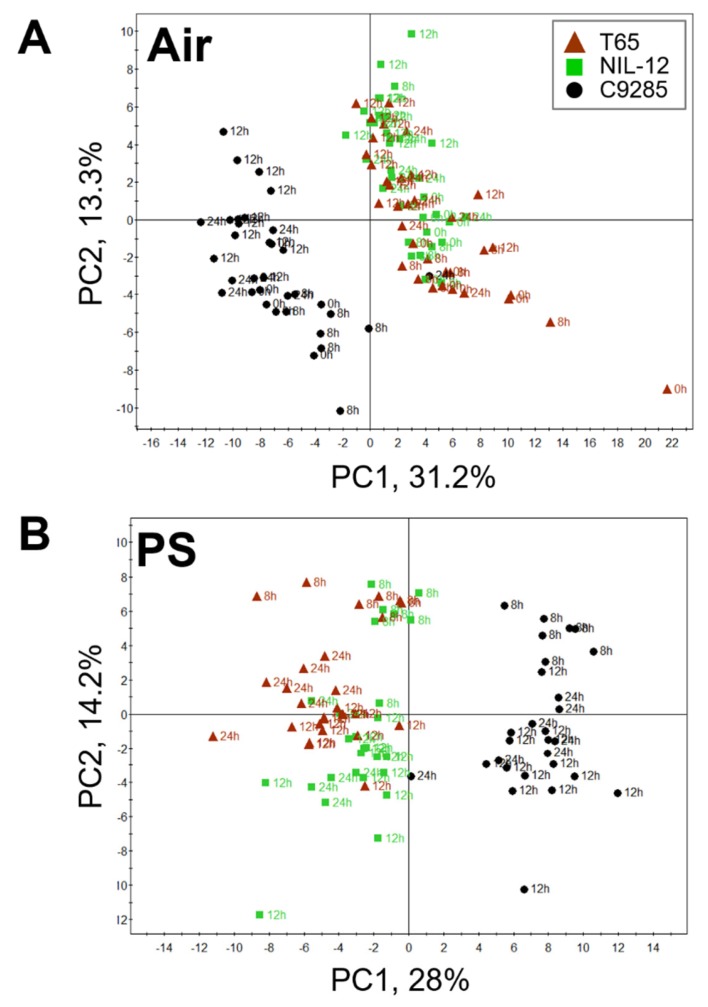
Overview of metabolite profiles and principal component analysis (PCA) score scatter plots of T65, NIL-12, and C9285 under Air (**A**) and PS conditions (**B**). We detected 283 peaks using GC-MS. Of these, 91 known metabolite peaks were identified or tentatively annotated, as well as 28 mass spectral tags (MSTs) [44,45]. The score scatter plot showed clear separation among the groups. Air, growth condition without submergence; PS, growth conditions by partial submergence treatment. Biological replicates, *n* = 7 except for time point 12 h (*n* = 14).

We measured shoot height and leaf sheath length of T65, NIL-12, and C9285 (Supplementary Figure S4) and confirmed induction of *SK1/2* expression in both NIL-12 and C9285 in the short-term PS treatment (Supplementary Figure S5). GC-MS-based metabolite profiling of T65, NIL-12, and C9285 detected 283 metabolite peaks in the short-term experiment. Of these, 91 known metabolite peaks were identified or tentatively annotated as well as 28 mass spectral tags (MSTs) [44,45]. The metabolite profiles were summarized and visualized by principal component analysis (PCA) (Figure 2). The score scatter plots showed clear separation of the groups of C9285 samples from the two groups of T65 and NIL-12 in both conditions (Figure 2A,B). These findings suggest that overall metabolite profiles differed between the two genetic backgrounds between C9285 and T65.

**Figure 3:**
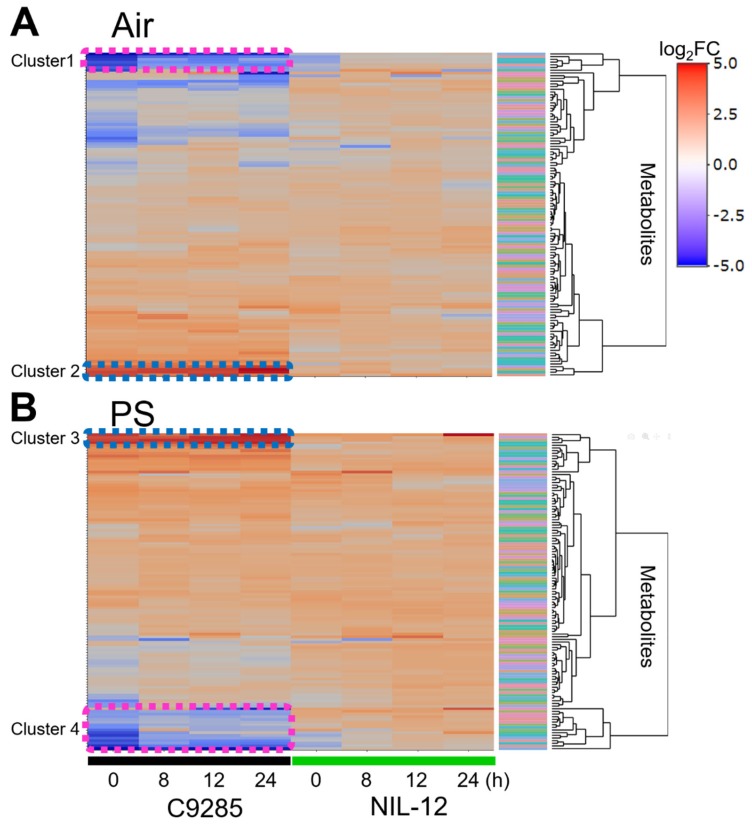
Hierarchical cluster analysis (HCA) of NIL-12, and C9285 against T65 under Air (**A**) and PS conditions (**B**). We used 119 metabolite peaks, including annotated metabolites and mass spectral tags (MSTs) [44,45]. The log_2_-fold change (FC) matrix for NIL-12 or C9285 versus T65 was used with Euclidean distance as similarity metrics and average linkage as the clustering method. Air, growth condition without submergence; PS, growth conditions by partial submergence treatment.

We used a pseudo-color heatmap of our metabolite profiles to explore the results further. Figure 3 shows the results of hierarchical cluster analysis (HCA) of NIL-12 and C9285 against T65 using fold-change values (see also Supplementary Data S1). C9285 accumulated metabolites associated with osmolytes, such as raffinose and galactinol, and phenylpropanoids, including caffeate and quinate conjugates (Clusters 2 and 3 in Figure 3). These osmolytes in C9285 plants were also greatly increased, even under Air conditions. In contrast, metabolite levels of five amino acids (i.e., asparagine, arginine, homoserine, methionine, and histidine) in C9285 were decreased over time compared to T65 under both Air and PS conditions (Clusters 1 and 4 in Figure 3, Supplementary Data S1).

**Figure 4:**
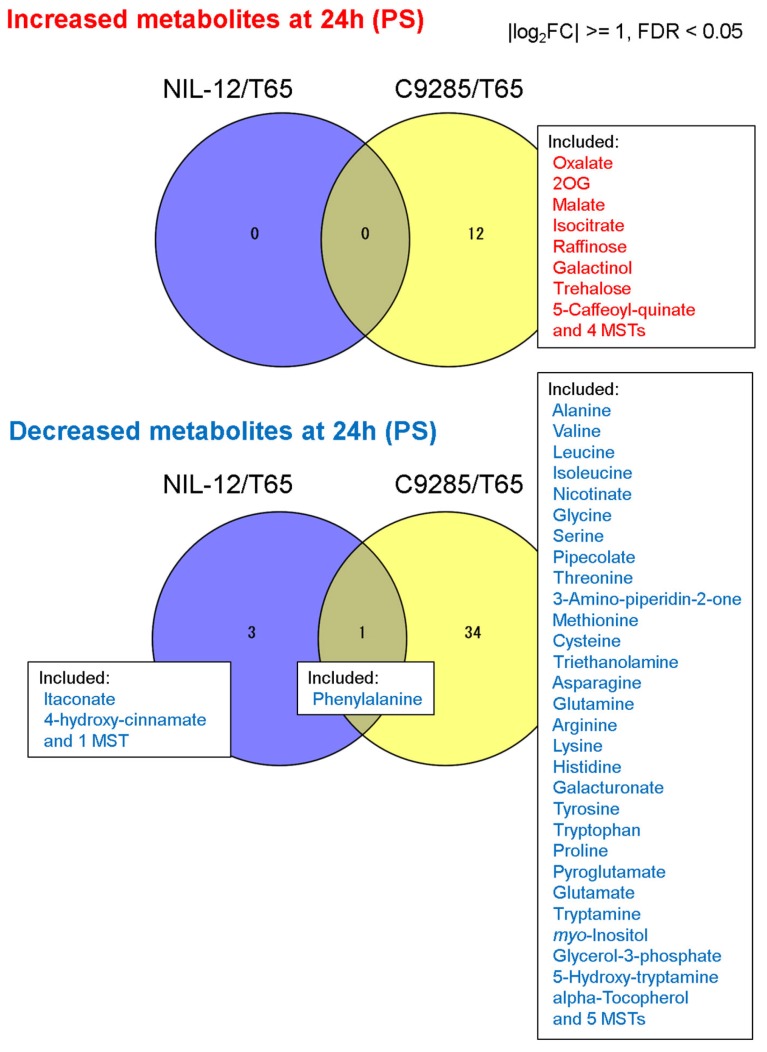
Venn diagrams of metabolites under PS treatment in the short-term experiment at 24 h (|log_2_FC| >= 1, FDR < 0.05). The number of metabolites that increased or decreased is shown. FC, fold-change; FDR, false discovery rate; 2OG, 2-oxoglutarate; MST, mass spectral tag [44,45].

In addition, the metabolite profiles of NIL-12 were nearly unchanged to those of T65 under the Air condition. In contrast, under PS conditions, we noted decreases in several primary metabolites, like amino acids and intermediates in the tricarboxylic acid (TCA) cycle, in both C9285 and NIL-12 plants (Supplementary Table S1). Figure 4 shows Venn diagrams of altered metabolite levels at 24 h following PS treatment in the short-term experiment [|log_2_Fold-change (FC)| >= 1, false discovery rate (FDR) < 0.05]. Compared to T65 plants, NIL-12 plants showed no significant increase in primary metabolites over time (Supplementary Figures S6–S8). The Venn diagram analysis also shows 3–18 significantly decreased metabolites in NIL-12 in comparison to T65.

In contrast, C9285 plants showed 12–16 metabolites significantly increased throughout the assayed conditions in the short-term experiment (Figure 4, Supplementary Figures S6–S8), such as galactinol, raffinose, and 5-caffeoyl-quinate. C9285 plants also showed significantly increased trehalose and malate levels at 8, 12, and 24 h after PS treatments. We identified 24–34 metabolites that decreased in C9285 plants. The 13 metabolites included valine, leucine, isoleucine, nicotinate, pipecolate, aspartate, glutamine, ornithine, lysine, histidine, and three MSTs (Supplementary Figure S6). We also observed that levels of 14 amino acids (i.e., tyrosine, tryptophan, threonine, serine, pyroglutamate, proline, phenylalanine, methionine, glycine, glutamate, γ-aminobutyric acid (GABA), cysteine, arginine, and alanine) and some metabolites (i.e., *myo*-inositol, urea, tryptamine, glycerol-3-phosphate, 5-hydroxy-tryptamine, and 3-amino-piperidin-2-one) decreased over the course of the experiment in C9285 plants. We also noticed that almost all the metabolites decreased under Air conditions (Supplementary Figure S6), suggesting direct or indirect reflection from subspecies effects. We observed decreased levels of branched chain amino acids, lysine, β-Alanine, putrescine, ornithine, nicotinate, triethanolamine, α-tocopherol, and pipecolate in the PS treatment from C9285 plants.

**Figure 5:**
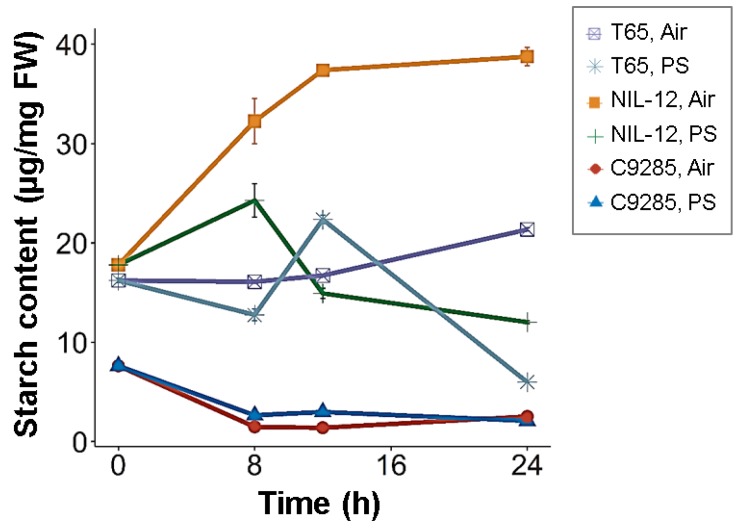
Quantification of starch content of T65, NIL-12, and C9285. We quantified starch content by the starch hydrolysis method [46]. Each point represents average starch content. Error bars represent standard deviation. Air, growth condition without submergence; PS, growth conditions by partial submergence treatment; FW, fresh weight. Biological replicates, *n* = 3.

We next quantified the starch content of T65, NIL-12, and C9285 to estimate the energy source of internode elongation in rice under PS conditions (Figure 5). Starch content was dramatically reduced at 8–24 h after PS treatment for NIL-12 compared to the Air condition. The starch levels in C9285 were lower than those of T65 and NIL-12 plants. Starch content of T65 and C9285 was unchanged in both Air and PS conditions.

### 2.3. Metabolite and Phytohormone Profiling of NIL-12 in the Long-Term PS Treatment

We measured the shoot height and leaf sheath length in NIL-12 and T65 plants during the long-term experiment (Supplementary Figure S9). Significant shoot height and leaf sheath length induction (Welch’s *t*-test, *p* < 0.05) was observed in NIL-12 and T65 at 72 h after the PS treatment. Shoot height at 288 h under PS conditions were 76.8 and 93.9 cm for T65 and NIL-12 plants, respectively. The length of the leaf sheath of T65 and NIL-12 plants was 34.6 and 49.2 cm under the same conditions. We noticed that PS treatments in both NIL-12 and T65 plants resulted in higher shoot and leaf sheath length than Air conditions. This observation suggests that PS might not be a severe stress condition compared to CS treatment, promoting their growth even in T65 plants (Supplementary Figure S9).

To investigate long-term metabolomic changes of T65 and NIL-12 samples under Air and PS conditions, multi-platform metabolomic analysis [42,43] was performed to capture broad metabolic and phytohormone changes. The resulting data matrix was summarized with non-redundant metabolite peaks with the MetMask [47], resulting in a metabolite data matrix consisting of 177 metabolites that included phytohormones and 48 samples (Supplementary Figure S10).

**Figure 6:**
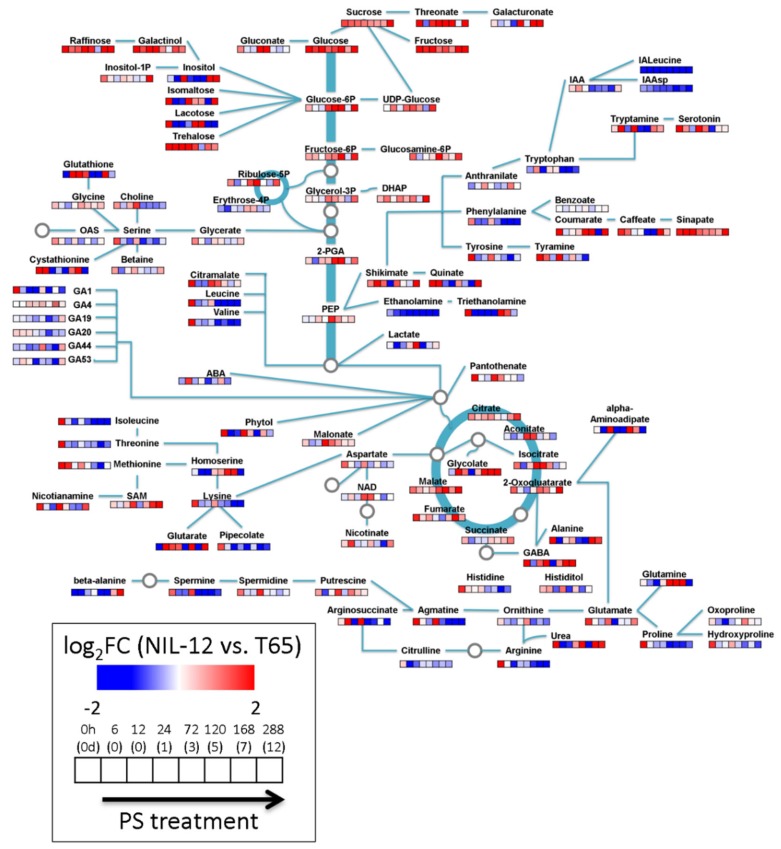
Pathway projection of metabolite and phytohormone profiles. The log_2_-transformed fold change (log_2_FC) is visualized by color: red, increased in NIL-12 plants by PS treatment; blue, decreased by the treatment. PS, growth conditions by partial submergence treatment. Biological replicates, *n* = 3.

**Figure 7:**
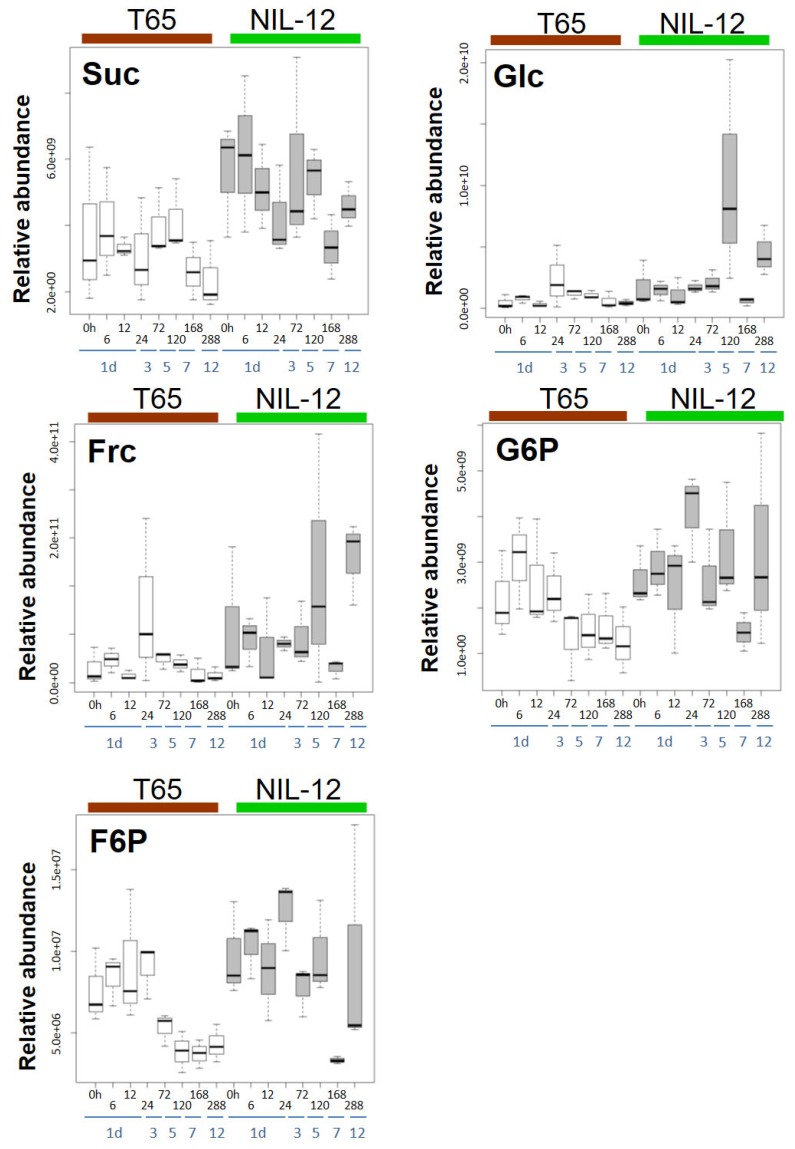
Changes of sugars and sugar phosphates during PS treatment. Boxplots represent metabolite relative abundances, indicating a data distribution using a five number summary (i.e., minimum, first quartile, median, third quartile, and maximum). Suc, sucrose; Glc, glucose; Frc, fructose; G6P, glucose 6-phosphate; F6P, fructose 6-phosphate. Biological replicates, *n* = 3.

**Figure 8:**
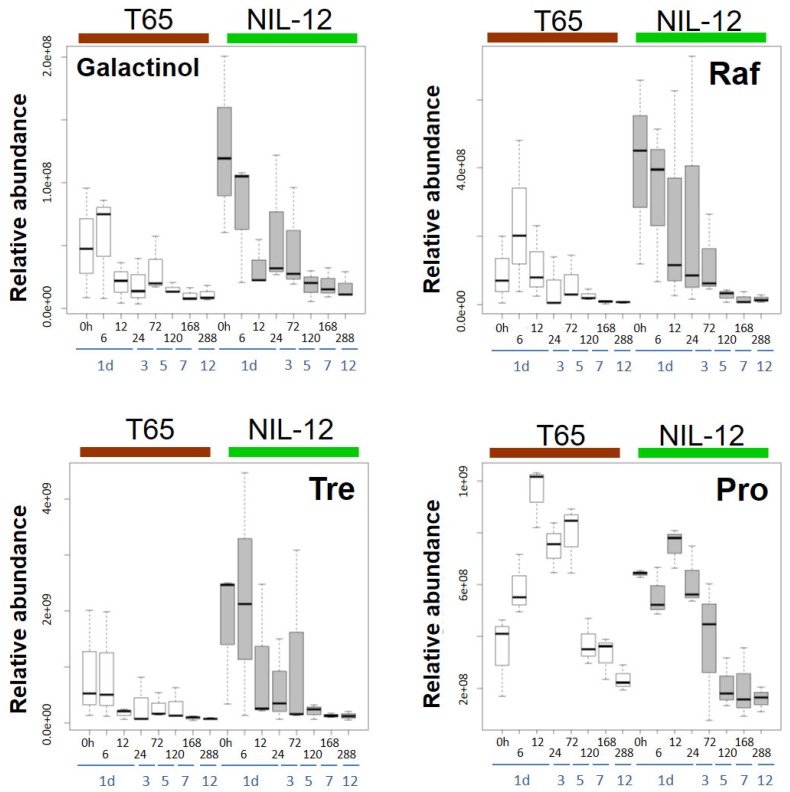
Changes of osmolytes during PS treatment. Boxplots represent the metabolite relative abundances, indicating data distributions using a five number summary (i.e., minimum, first quartile, median, third quartile, and maximum). Raf, raffinose, Tre, trehalose; Pro, proline. Biological replicates, *n* = 3.

**Figure 9:**
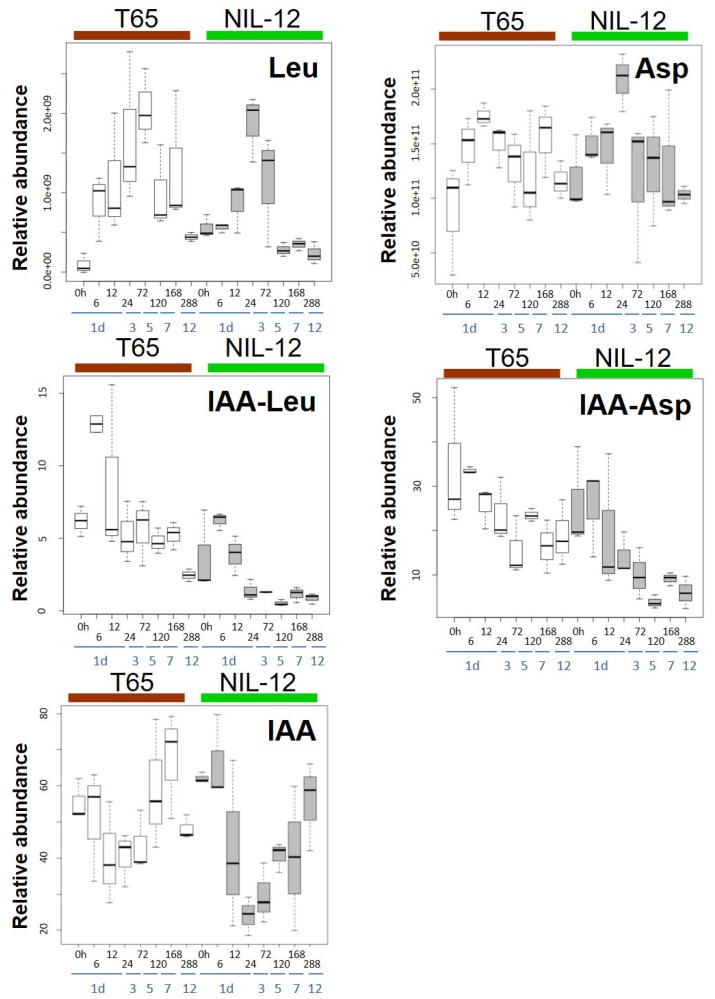
Changes of indole-3-acetic acid (IAA), IAA conjugates, and their substrates during PS treatment. Boxplots represent metabolite relative abundances, indicating data distributions using a five number summary (i.e., minimum, first quartile, median, third quartile, and maximum). IAA-Leu and IAA-Asp, indole-3-acetate (IAA) conjugates with leucine and aspartate, respectively. Biological replicates, *n* = 3.

Metabolite and phytohormone profiles were overlaid on metabolite pathways (Figure 6). The metabolite profiles of NIL-12 samples showed enhanced levels of metabolites involved in glycolysis compared to T65 samples (Figure 6 and Figure 7) as well as increases in levels of sugar-derived osmolytes, i.e., trehalose, galactinol, and raffinose (Figure 8). The levels of amino acids, including branched chain amino acids, lysine, threonine, arginine, proline, phenylalanine, and tryptophan, decreased dramatically in NIL-12 after the PS treatment (Figure 6, Figure 8, Figure 9, and Supplementary Figure S11). However, there were no significant changes of other hypoxia-inducible metabolites, i.e., alanine, GABA, glutamine, and glutamate (see [38,48]) (Supplementary Figure S11). These metabolomic fluctuations were NIL-12 specific, rather opposite changes, when compared to those that have been reported in flash-flood tolerant rice [37,38,39]. After the PS treatment, two conjugates of indole-3-acetic acid (IAA) with leucine and aspartate content (i.e., IAA-Leu and IAA-Asp) were decreased in NIL-12 over time (Figure 9). IAA and other phytohormone content (abscisic acid (ABA) and gibberellins (GAs)) did not differ when compared to T65 (Figure 6 and Figure 9).

## 3. Discussion

This study investigated broad range metabolic and hormonal changes to identify key pathways in relation to flooding stress in deepwater rice. We assessed metabolic responses in NIL-12 [23,25] to focus on the impact of the major QTL on chromosome 12 that includes the *SNORKEL* genes [25], using multi-platform metabolomics approaches [42,43]. In this study, 81 peaks from GC-MS, 77 from CE-MS, and 11 from LC-MS-based phytohormone analyses were detected (Supplementary Figure S10). Comparative metabolome analysis using GC-MS and NMR supported the previous finding that *SUB1A* was responsible for regulating carbohydrate levels during submergence [37,38]. The present study suggests that a multi-platform metabolome approach can compensate for low detection coverage by a single platform (for example, see [48]).

We first evaluated whether light and submergence treatments affected internode elongation of C9285 (Figure 1). TIL and shoot height showed the same pattern, indicating that shoot elongation was due to internode elongation of C9285 (Figure 1A,B). TIL increased under PS and CS conditions. On the other hand, under PD conditions, shoot height and TIL increased until four days after submergence, then these lengths did not change. Samples after 10 days of CD died (Figure 1C). These results suggest that the presence of light was necessary to elongate internodes during long-term submergence. The SPAD value that reflects the relative leaf chlorophyll content decreased after 10 days under CS conditions, though the extent of TIL was similar between PS and CS treatments. These findings imply that C9285 may have different strategies to keep appropriate photosynthesis under PS conditions during long-term submergence. Naturally, flooding reduces photosynthesis via impedance of gas exchange and restricted accession to CO_2_. Re-establishment of aerial contact can recover some photosynthesis due to the escape strategy. Rice, as well as many other plants, has superhydrophobic leaf surfaces, which can maintain CO_2_ and O_2_ exchange with the surrounding environments. The leaf gas films contribute to submergence tolerance in rice plants by allowing underwater gas exchange [49,50]. Kurokawa and colleagues identified the gene *Leaf Gas Film 1* (*LGF1*) that contributes to the regulation of plant gas exchange under submerged conditions [51]. The evaluation of leaf gas film retention due to submergence in both deepwater- and paddy rice plants showed that leaf gas films did not persist beyond six to eight days of submergence [52]. The reduced oxygen availability during submergence causes a wide range of metabolic reprogramming in plants (for example, see [53]). There are several metabolites [38,48] (Supplementary Figure S11) that showed significant increases in rice near-isogenic line M202(*Sub1*) variety by complete submerged treatments [54]. In Arabidopsis roots exposed to anoxic conditions, accumulation of proline, GABA, and alanine was observed [55]. Waterlogging and hypoxia also affect the glycolysis pathway and TCA cycle [39]. Metabolomic approaches using highly flood-tolerant *Lotus japonicus* plants suggested the importance of alanine metabolism, of which, alanine amino transferase links glycolysis with the TCA cycle during hypoxia [56]. For example, metabolite profiling of *Lotus japonicus* showed that alanine, GABA, and glutamine over-accumulated, while TCA cycle intermediates decreased during waterlogging. These metabolite levels behaved differently in C9285 and NIL-12 plants (Figure 4, Figure 6, and Supplementary Figure S6–S8). Starch, sucrose, glucose, and fructose content increased in NIL-12 during PS conditions (Figure 5 and Figure 6), while most of the sugars were already increased at Air conditions (0 h), especially sucrose, which might be one reason for the fast increasing starch content at PS treatment. In the case of M202(*Sub1*), significant depletion of starch and carbohydrates was observed when compared to control plants under CS conditions [37,57]. These results may reflect the different metabolomic strategies of *SUB1* [19,20] or *SK1/2* [25] loci in terms of metabolic perturbations during the two types of flooding.

Under Air condition, the levels of eight annotated metabolites were significantly increased in the profiles of C9285 against T65. However, such metabolite changes were not observed in metabolite comparison between NIL-12 and T65 under the same condition (Supplementary Figure S6). It might be a reflection of metabolomic differences between different subspecies, i.e., *Indica* and *Japonica* rice [28]. We compared metabolite changes of NIL-12 and T65 in short- and long-term experiments at the same sampling points (Supplementary Tables S1 and S2). At Air condition, 13 annotated metabolite levels in NIL-12 plants were decreased in the short-term experiment, while there were no metabolites that were significantly increased. On the other hand, 16 metabolite levels in NIL-12 plants were increased in the long-term experiment, while there were no metabolites that were significantly decreased. One of the possibilities why such a contradiction was observed at the Air condition might be the harvested year-derived difference. PS treatments of NIL12 and T65 within 24 h in short- and long-term experiments led to a decrease in metabolite levels, including amino acids. Amino acid consumption was also observed during PS in C9285 and NIL-12 when compared to T65 (Figure 4 and Figure 6, Supplementary Figures S6–S8), although amino acid levels in rice and *Lotus japonicus* increased in response to prolonged CS [38,56]. As the levels of amino acids in these plants decreased after de-submergence, these amino acids probably were used for shoot growth, like in deepwater rice [56,58].

Although *SK1/2* [25] are positive regulators of rapid internode elongation in deepwater rice, the downstream direct targets regulated by *SK1/2* remain unclear. Active gibberellin biosynthesis plays an important role in deepwater responses [27,28]. Uncharacterized loci on chromosome 3 in deepwater rice, along with *SK1/2* [25] and *SD1* [33], appear necessary to explain the full deepwater response [23]. The following phytohormones also are associated with modulation of underwater elongation growth: ethylene as a trigger, ABA a repressor, and GA/auxin promoters [2,59,60]. In NIL-12, active gibberellins, GA_1_ and GA_4_, were unchanged after PS treatment compared with T65 (Figure 6). Both GA_1_ and GA_4_ content were increased in C9285 nodes after 6 h of PS treatment [25,28]. These trends were also observed in NIL-1, possessing C9285 segments for the QTL on chromosomes 1 in the T65 genetic background [33]. Our result supports that the increase of active GAs in C9285 under submergence depends on chromosome 1 but not on chromosome 12 QTL [33]. Since the internode elongation ratio of NIL-12 could explain approximately 40% of C9285 [25], unchanged active GA levels in NIL-12 observed in the study might be explained by the regulation system of phytohormone sensitivity and/or presence of other factors to induce internode elongation under PS conditions. One possible explanation is the contribution of metabolites related to stress response (Figure 4, Figure 6, Figure 8, and Figure 9). Our metabolomic approach led to the identification of osmolytes as possible key players for the complex deepwater response (Figure 4 and Figure 6), although these metabolites were already increased under Air conditions. This observation might be a difference related to the subspecies and might be a preadaptation to stress conditions present in C9285 compared to T65 plants.

In this study, the IAA level was similar between T65 and NIL-12 during PS conditions (Figure 9). On the other hand, we identified decreased auxin conjugates, like IAA-Leu and IAA-Asp in NIL-12, when compared to those in T65 [61,62] (Figure 9). The levels of these conjugates are known to maintain auxin homeostasis during stress and defense responses [62]. IAA-Leu and IAA-Asp are inactive forms of IAA. On these, IAA-Asp is catabolized, while IAA-Leu is stored forms of IAA that can be accessed at a later time [63,64]. Increase of IAA-Asp can contribute abiotic stress tolerance in plants [64,65,66], though there are few sources of information about the association between IAA-Leu content and abiotic stress tolerance. The lower levels of IAA-Asp and IAA-Leu in NIL-12 than those in T65 might explain different stress status between NIL-12 and T65.

Our study highlighted a wide range of short and long-term metabolic responses to PS stress in deepwater rice, implying that our metabolome and hormonome information will serve as an important data resource for further investigations of targeted metabolism along with different types of flooding stress responses—escape or quiescent strategies—in rice plants.

## 4. Materials and Methods

### 4.1. Plant Material and Cultivation

A deepwater rice (*Oryza sativa*) cultivar (cv. C9285, *indica-japonica* admixed) and a non-deepwater rice cultivar (cv. T65, *japonica*) were used in this study. T65 was maintained at Nagoya University; C9285 was provided by the National Institute of Genetics in Japan. The NIL-12 possessing C9285 segments for the deepwater response QTL on chromosomes 12 in the T65 genetic background was described previously [23]. Rice seeds were incubated at 60°C for 10 min followed by pre-germination at 28°C in water for 3 to 4 d. Afterward, germinated seeds were transferred to plastic pots containing soil mixture (Mikawa Baido; AICHI Mederu, Nishio, Japan) and grown in the greenhouse in Nagoya, Japan, in June under natural light cycles of approximately 14 h light/10 h dark.

### 4.2. Submergence Treatments

For submergence treatments of deepwater rice under different light and submergence conditions, we treated C9285 at the 9–10 leaf stage in tanks (diameter = 120 cm, height = 160 cm) under four combinations: partial submergence (PS) with light, complete submergence (CS) with light, PS with darkness (PD), and CS with darkness (CD). The four treatments were employed to evaluate growth effects and chlorophyll contents for C9285. For PD and CD, we used a light shielding cloth (Supplementary Figure S1). TIL was scored as the total length of internodes longer than 5 mm. The length of the leaf sheath was defined as the length of the most elongated leaf sheath in each seedling. Chlorophyll content was analyzed using a SPAD chlorophyll meter SPAD-502Plus (Konica Minolta, Tokyo, Japan).

For the short-term submergence treatment, rice seedlings at the 9–10 leaf stage were completely submerged for 0, 8, 12, and 24 h. To avoid differences in gene expression between samples due to circadian rhythms, submergence treatments were initiated at different times of the day and all samples collected in the morning for the short-term experiment. We also harvested samples at the 8–10 leaf stage for a long-term experiment (0, 6, 12, 24, 48, 72, 120, 168, and 288 h). After the submergence treatment, 5 mm of the shoot base region containing internodes, nodes, the shoot apex, and basal regions of leaves were sampled, rapidly frozen in liquid nitrogen, and stored at −80 °C until further analyses.

### 4.3. Semi-Quantitative RT-PCR Analysis

Semi-quantitative RT-PCR analysis of the *SK1/2* genes was performed with rice samples derived from the same plant parts and time points as the short-term experiment described above. Total RNA was extracted using RNeasy Plant Mini Kit (Qiagen) with RNase-free DNase I (Qiagen), according to the manufacturer’s protocol. First-strand cDNA was synthesized with the Omniscript RT Kit (Qiagen) with oligodT-20 primers. The semi-quantitative RT-PCR was performed with Ex Taq polymerase (Takara Bio, Otsu, Japan) in a reaction volume of 25 μL, according to the manufacturer’s protocol. The *OsACT1* (LOC_Os03g50885) gene was used as an internal control. The gene specific primers were as follows: TK638 (5′-ACGGTATCCCTGAACTACTG-3′) and TK639 (5′-TCGTAGCGACAGCCGTACTG-3′) for *SK1*, TK640 (5′-CACTGGAGGCAACGAATG-3′) and TK641 (5′-TAAAAGGACCAGAGGCAGC-3′) for *SK2*, OsACT1-F (5′-TCCATCTTGGCATCTCTCAG-3′) and OsACT1-R (5′-GTACCCTCATCAGGCATCTG-3′) for *OsACT1*.

### 4.4. Starch Measurement

Powdered leaf tissue (about 100 mg fresh weight (FW)) was soaked in 200 µL of 0.7 M perchloric acid kept at 0 °C. After centrifugation at 3000× *g* for 10 min, the pellet was collected and washed twice in 300 µL of cold distilled water. The pellet was resuspended in 200 µL of 0.1 N NaOH, and boiled for 2 min. After neutralization with 20 µL of 3 M Na-acetate buffer (pH 5.0), the starch sample was digested with 6.7 units amyloglucosidase (Toyobo, Osaka, Japan) in 500 µL of 5 mM sodium acetate buffer (pH 4.4) for 1 h at 40 °C. The reaction was stopped by boiling for 10 min. After centrifugation at 15,000× *g* for 20 min, a quantitative glucose assay was conducted according to Bergmeyer et al. [46]. The _D_-glucose in the supernatant was measured by the increase in absorbance at 340 nm in the 0.17 U of Hexokinase and 0.085 U of Glucose-6-Phosphate Dehydrogenase (Sigma-Aldrich, Tokyo, Japan) after mixing in a 160 μL of a solution consisting of 60 mM HEPES-NaOH (pH 7.4), 5 mM MgCl_2_, 2 mM NADP, and 25 mM ATP. The amount of glucose in the range from 0 to 10 µg was determined. Starch content was estimated by multiplying the glucose equivalent of 0.9.

### 4.5. GC-MS Analysis

Metabolite profiling with gas chromatography-time-of-flight-mass spectroscopy (GC-MS) was conducted as described in [67,68]. Equivalent of 10 mg FW sample was used for the short- and the long-term experiments. Samples were subjected to derivatization, and then the derivatized samples were injected into the GC-MS instrument for detection of central metabolites and intermediates of secondary metabolites. The chromatograms were pre-processed using hyphenated data analysis (HDA) and raw data analysis (RDA) [69,70] and then normalized using the CCMN algorithm [71].

### 4.6. CE-MS Analysis

Metabolite profiling with a capillary electrophoresis-time-of-flight-mass spectroscopy (CE-MS, cation and anion mode) was performed according to [72]. We used the equivalent of 40 mg FW sample for the analysis.

### 4.7. Measurement of Hormone Levels

Phytohormone analysis was performed as previously described [73]. Briefly, 200 mg FW samples were used for the analysis. Quantification was conducted with a UHPLC-ESI-qMS/MS (AQUITY UPLC™ System/Quattro Premier XE, Waters, Milford, MA, USA) with an octa decyl silyl (ODS) column (AQUITY UPLC BEH C_18_ 1.7 µm, 2.1 × 100 mm, Waters). For quantification of IAA and IAA derivatives, GA, ABA, and cytokinins (CK) contents, the FW of each plant sample was determined.

### 4.8. Data Analysis

To identify differentially accumulated metabolites between deepwater- (C9285 or NIL-12) and non-deepwater rice T65 or among time points, we calculated log_2_-mean expression fold-change of each metabolite in C9285 (or NIL-12) compared to T65. We utilized the R package LIMMA [74,75] with false discovery rate (FDR) correction for multiple testing [76]. PCA was applied for the metabolite data matrix (log_10_-transformed) with autoscaling using SIMCA-P + (v 13.0, Umetrics, Sweden) software. HCA and visualization of the metabolite levels (log_2_ ratio) were performed in R (https://cran.r-project.org/) and the hetmaply package [77]. Euclidean distances in stats::dist() and the complete linkage method in stats::hclust() were used for HCA. For the short-term experiment, we analyzed plant samples on two different days and normalized them with the COMBAT algorithm [78] to remove potential batch effects. In the long-term experiment, we combined and summarized metabolome and phytohormonome data obtained from different platforms using the MetMask tool [47]. VENNY (https://bioinfogp.cnb.csic.es/tools/venny/index.html) was used to create the Venn diagram.

## 5. Conclusions

Rice is an important staple crop in the flood-prone regions of Asia and West Africa [8]. In these areas, flooding is a common natural disaster, which greatly reduces rice yield. Deepwater rice varieties are grown mainly in Asian lowland areas and can respond to deep flooding by enhancing rapid internode elongation as water levels rise. Key factors like *SNORKEL* and *SD1* genes contribute to this important trait [25,33]. In this study, we evaluated the metabolic responses to flooding stress in non-deepwater T65 and deepwater rice cultivars, C9285, and NIL-12 plants, using a multi-platform metabolomics approach [42,43]. Our analysis identified key osmolytes, metabolites in glycolysis, and the TCA cycle, as well as auxin conjugates, as important components of the deepwater stress response.

## Supplementary Materials

The following are available online at https://www.mdpi.com/2218-1989/10/2/68/s1, Figure S1. Conditions of submergence treatments under light–dark conditions. (A) Schematic overview of each treatment. Percentages are based on a ratio of shoot height to water level. Air, water level is under the soil surface; PL, PS treatment under light condition; CL, CS treatment under light condition; PD, PS treatment under dark condition; and CD, CS treatment under dark condition. (B) A tank for submergence treatment under light conditions. (C) A tank covered with light shielding cloth for submergence treatment under dark conditions. Abbreviations: PS, growth conditions by partial submergence treatment; CS, complete submergence, Figure S2. Conditions of partial submergence treatments in the greenhouse. (A) Air condition. Water level is under the soil surface. (B) Partial submergence condition, 70% of aerial parts were soaked in water, Figure S3. Experimental design of short-term- (A) and long-term (B) experiments. The orange arrow represents sampling points under Air conditions, while the blue arrow represents sampling points under PS conditions. Abbreviations: Air, growth condition without submergence; PS, growth conditions by partial submergence treatment. *In the short-term experiment, internodes were sampled at 8 h after treatment, Figure S4. Length of shoot and leaf sheath (LS) of T65, NIL-12, and C9285 in the short-term experiment. Shoot height (A) and LS length (B) were measured after Air or PS treatments. Boxplots represent metabolite relative abundances. Asterisks show differences from the Air 0 h condition (Welch’s *t*-test, * *p* < 0.05). Abbreviations: Air, growth condition without submergence; PS, growth conditions by partial submergence treatment. Biological replicates, *n* = 6, Figure S5. Semi-quantitative RT-PCR analysis of *SK1/2* genes in T65, NIL-12, and C9285. T65, NIL-12, and C9285 plants at the 9–10-leaf stage were subjected to each condition. Semi-quantitative RT-PCR was performed with 35, 35, and 25 cycles for *SK1*, *SK2*, and *OsACT1*, respectively. *OsACT1* (rice Actin1 gene) was used for an internal standard control. Air, growth condition without submergence; PS, growth conditions by partial submergence treatment, Figure S6. Venn diagrams of altered metabolites in the short-term experiment at 0 h (Air) (|log_2_FC| >= 1, FDR < 0.05). The number of increased and decreased metabolites is shown. FC, fold-change; FDR, false discovery rate; 2OG, 2-oxoglutarate; GABA, γ-aminobutyric acid; MST, mass spectral tag [44,45], Figure S7. Venn diagrams of altered metabolites in the PS treatment in the short-term experiment at 8 h (|log_2_FC| >= 1, FDR < 0.05). The number of increased and decreased metabolites is shown. FC, fold-change; FDR, false discovery rate; PS, partial submergence; GABA, γ-aminobutyric acid; MST, mass spectral tag [44,45], Figure S8. Venn diagrams of altered metabolites in the short-term experiment at 12 h (PS) (|log_2_FC| >= 1, FDR < 0.05). The number of increased and decreased metabolites is shown. FC, fold-change; FDR, false discovery rate; PS, partial submergence; 2OG, 2-oxoglutarate; MST, mass spectral tag MSTs [44,45], Figure S9. Length of shoot and leaf sheath (LS) of NIL-12 and T65 in the long-term experiment. Shoot height (A) and LS length (B) were measured after Air or PS treatments. Boxplots represent shoot and LS length. Asterisks represent statistically significant differences from the Air 0 h condition (Welch’s *t*-test, * *p* < 0.05). Air, air conditions; PS, growth conditions by partial submergence treatment. Biological replicates, *n* = 4–8, Figure S10. Data summarization of the metabolite and phytohormone dataset. We used metabolite and phytohormone profiles after summarizing with the MetMask tool [47] for statistical analysis. Abbreviations: GC, GC-MS analysis; and CE, CE-MS analysis, Figure S11. Changes of known hypoxia-inducible metabolites described in Barding et al. (2012) [38] and Ruperti et al. (2019) [48]. Boxplots represent the metabolite relative abundances. Air, growth condition without submergence; PS, growth conditions by partial submergence treatment; Ala, Alanine; GABA, γ-aminobutyric acid; Ile, isoleucine; Thr, threonine; Val, valine; Gln, glutamine; Glu, glutamate. Biological replicates, *n* = 3, Table S1. Summary of metabolome data matrix, statistical data analysis, and phenodata of T65, C9285, and NIL-12 in short-term experiment, Table S2. Summary of metabolome data matrix, statistical data analysis, and phenodata of T65 and NIL-12 in long-term experiment, Data S1. Zip-compressed files for the inspection of metabolite profiles in short-term experiments by interactive heatmaps. These are based on the heatmaply package [77].

## References

[1] Bailey-Serres J., Voesenek L.A. (2008). Flooding stress: Acclimations and genetic diversity. Annu. Rev. Plant Biol..

[2] Hattori Y., Nagai K., Ashikari M. (2011). Rice growth adapting to deepwater. Curr. Opin. Plant Biol..

[3] Akman M., Bhikharie A.V., McLean E.H., Boonman A., Visser E.J., Schranz M.E., van Tienderen P.H. (2012). Wait or escape? Contrasting submergence tolerance strategies of Rorippa amphibia, Rorippa sylvestris and their hybrid. Ann. Bot..

[4] Voesenek L.A., Bailey-Serres J. (2015). Flood adaptive traits and processes: An overview. New Phytol..

[5] Loreti E., van Veen H., Perata P. (2016). Plant responses to flooding stress. Curr. Opin. Plant Biol..

[6] Nagai K., Hattori Y., Ashikari M. (2010). Stunt or elongate? Two opposite strategies by which rice adapts to floods. J. Plant Res..

[7] Vriezen W.H., Zhou Z., Van Der Straeten D. (2003). Regulation of submergence-induced enhanced shoot elongation in *Oryza sativa* L.. Ann. Bot..

[8] Singh A., Septiningsih E.M., Balyan H.S., Singh N.K., Rai V. (2017). Genetics, Physiological Mechanisms and Breeding of Flood-Tolerant Rice (*Oryza sativa* L.). Plant Cell Physiol..

[9] Vreeburg R.A., Benschop J.J., Peeters A.J., Colmer T.D., Ammerlaan A.H., Staal M., Elzenga T.M., Staals R.H., Darley C.P., McQueen-Mason S.J. (2005). Ethylene regulates fast apoplastic acidification and expansin A transcription during submergence-induced petiole elongation in *Rumex palustris*. Plant J..

[10] Pierik R., van Aken J.M., Voesenek L.A. (2009). Is elongation-induced leaf emergence beneficial for submerged Rumex species?. Ann. Bot..

[11] Van Veen H., Mustroph A., Barding G.A., Vergeer-van Eijk M., Welschen-Evertman R.A., Pedersen O., Visser E.J., Larive C.K., Pierik R., Bailey-Serres J. (2013). Two Rumex species from contrasting hydrological niches regulate flooding tolerance through distinct mechanisms. Plant Cell.

[12] Herzog M., Pedersen O. (2014). Partial versus complete submergence: snorkelling aids root aeration in Rumex palustris but not in *R. acetosa*. Plant Cell Environ..

[13] Sasidharan R., Mustroph A., Boonman A., Akman M., Ammerlaan A.M., Breit T., Schranz M.E., Voesenek L.A., van Tienderen P.H. (2013). Root transcript profiling of two Rorippa species reveals gene clusters associated with extreme submergence tolerance. Plant Physiol..

[14] Akman M., Bhikharie A., Mustroph A., Sasidharan R. (2014). Extreme flooding tolerance in Rorippa. Plant Signal. Behav..

[15] Estioko L.P., Miro B., Baltazar A.M., Merca F.E., Ismail A.M., Johnson D.E. (2014). Differences in responses to flooding by germinating seeds of two contrasting rice cultivars and two species of economically important grass weeds. AoB Plants.

[16] Nah G., Im J.H., Kim J.W., Park H.R., Yook M.J., Yang T.J., Fischer A.J., Kim D.S. (2015). Uncovering the differential molecular basis of adaptive diversity in three *Echinochloa leaf* transcriptomes. PLoS ONE.

[17] Nandi S., Subudhi P.K., Senadhira D., Manigbas N.L., Sen-Mandi S., Huang N. (1997). Mapping QTLs for submergence tolerance in rice by AFLP analysis and selective genotyping. Mol. Gen. Genet..

[18] Siangliw M., Toojinda T., Tragoonrung S., Vanavichit A. (2003). Thai jasmine rice carrying QTLch9 (SubQTL) is submergence tolerant. Ann. Bot..

[19] Xu K., Xu X., Fukao T., Canlas P., Maghirang-Rodriguez R., Heuer S., Ismail A.M., Bailey-Serres J., Ronald P.C., Mackill D.J. (2006). Sub1A is an ethylene-response-factor-like gene that confers submergence tolerance to rice. Nature.

[20] Fukao T., Yeung E., Bailey-Serres J. (2011). The submergence tolerance regulator SUB1A mediates crosstalk between submergence and drought tolerance in rice. Plant Cell.

[21] Tang D.Q., Kasai Y., Miyamoto N., Ukai Y., Nemoto K. (2005). Comparison of QTLs for Early Elongation Ability between Two Floating Rice Cultivars with a Different Phylogenetic Origin. Breed. Sci..

[22] Hattori Y., Miura K., Asano K., Yamamoto E., Mori H., Kitano H., Matsuoka M., Ashikari M. (2007). A Major QTL Confers Rapid Internode Elongation in Response to Water Rise in Deepwater Rice. Breed. Sci..

[23] Hattori Y., Nagai K., Mori H., Kitano H., Matsuoka M., Ashikari M. (2008). Mapping of three QTLs that regulate internode elongation in deepwater rice. Breed. Sci..

[24] Kawano R., Doi K., Yasui H., Mochizuki T., Yoshimura A. (2008). Mapping of QTLs for floating ability in rice. Breed. Sci..

[25] Hattori Y., Nagai K., Furukawa S., Song X.J., Kawano R., Sakakibara H., Wu J., Matsumoto T., Yoshimura A., Kitano H. (2009). The ethylene response factors SNORKEL1 and SNORKEL2 allow rice to adapt to deep water. Nature.

[26] Nagai K., Kuroha T., Ayano M., Kurokawa Y., Angeles-Shim R.B., Shim J.H., Yasui H., Yoshimura A., Ashikari M. (2012). Two novel QTLs regulate internode elongation in deepwater rice during the early vegetative stage. Breed. Sci..

[27] Nagai K., Kondo Y., Kitaoka T., Noda T., Kuroha T., Angeles-Shim R.B., Yasui H., Yoshimura A., Ashikari M. (2014). QTL analysis of internode elongation in response to gibberellin in deepwater rice. AoB Plants.

[28] Ayano M., Kani T., Kojima M., Sakakibara H., Kitaoka T., Kuroha T., Angeles-Shim R.B., Kitano H., Nagai K., Ashikari M. (2014). Gibberellin biosynthesis and signal transduction is essential for internode elongation in deepwater rice. Plant Cell Environ..

[29] Fukushima A., Kusano M., Redestig H., Arita M., Saito K. (2009). Integrated omics approaches in plant systems biology. Curr. Opin. Chem. Biol..

[30] Rai A., Saito K., Yamazaki M. (2017). Integrated omics analysis of specialized metabolism in medicinal plants. Plant J..

[31] Jung K.H., Seo Y.S., Walia H., Cao P., Fukao T., Canlas P.E., Amonpant F., Bailey-Serres J., Ronald P.C. (2010). The submergence tolerance regulator Sub1A mediates stress-responsive expression of AP2/ERF transcription factors. Plant Physiol..

[32] Minami A., Yano K., Gamuyao R., Nagai K., Kuroha T., Ayano M., Nakamori M., Koike M., Kondo Y., Niimi Y. (2018). Time-Course Transcriptomics Analysis Reveals Key Responses of Submerged Deepwater Rice to Flooding. Plant Physiol..

[33] Kuroha T., Nagai K., Gamuyao R., Wang D.R., Furuta T., Nakamori M., Kitaoka T., Adachi K., Minami A., Mori Y. (2018). Ethylene-gibberellin signaling underlies adaptation of rice to periodic flooding. Science.

[34] Saito K., Matsuda F. (2010). Metabolomics for functional genomics, systems biology, and biotechnology. Annu. Rev. Plant Biol..

[35] Sumner L.W., Lei Z., Nikolau B.J., Saito K. (2015). Modern plant metabolomics: Advanced natural product gene discoveries, improved technologies, and future prospects. Nat. Prod. Rep..

[36] Alseekh S., Fernie A.R. (2018). Metabolomics 20 years on: What have we learned and what hurdles remain?. Plant J..

[37] Barding G.A., Beni S., Fukao T., Bailey-Serres J., Larive C.K. (2013). Comparison of GC-MS and NMR for metabolite profiling of rice subjected to submergence stress. J. Proteome Res..

[38] Barding G.A., Fukao T., Beni S., Bailey-Serres J., Larive C.K. (2012). Differential metabolic regulation governed by the rice SUB1A gene during submergence stress and identification of alanylglycine by 1H NMR spectroscopy. J. Proteome Res..

[39] Locke A.M., Barding G.A., Sathnur S., Larive C.K., Bailey-Serres J. (2018). Rice SUB1A constrains remodelling of the transcriptome and metabolome during submergence to facilitate post-submergence recovery. Plant Cell Environ..

[40] Herzog M., Fukao T., Winkel A., Konnerup D., Lamichhane S., Alpuerto J.B., Hasler-Sheetal H., Pedersen O. (2018). Physiology, gene expression, and metabolome of two wheat cultivars with contrasting submergence tolerance. Plant Cell Environ..

[41] Wang X., Zhu W., Hashiguchi A., Nishimura M., Tian J., Komatsu S. (2017). Metabolic profiles of flooding-tolerant mechanism in early-stage soybean responding to initial stress. Plant Mol. Biol..

[42] Kusano M., Baxter I., Fukushima A., Oikawa A., Okazaki Y., Nakabayashi R., Bouvrette D.J., Achard F., Jakubowski A.R., Ballam J.M. (2015). Assessing metabolomic and chemical diversity of a soybean lineage representing 35 years of breeding. Metabolomics.

[43] Kusano M., Redestig H., Hirai T., Oikawa A., Matsuda F., Fukushima A., Arita M., Watanabe S., Yano M., Hiwasa-Tanase K. (2011). Covering chemical diversity of genetically-modified tomatoes using metabolomics for objective substantial equivalence assessment. PLoS ONE.

[44] Kopka J., Schauer N., Krueger S., Birkemeyer C., Usadel B., Bergmuller E., Dormann P., Weckwerth W., Gibon Y., Stitt M. (2005). GMD@CSB.DB: The Golm Metabolome Database. Bioinformatics.

[45] Schauer N., Steinhauser D., Strelkov S., Schomburg D., Allison G., Moritz T., Lundgren K., Roessner-Tunali U., Forbes M.G., Willmitzer L. (2005). GC-MS libraries for the rapid identification of metabolites in complex biological samples. FEBS Lett..

[46] Bergmeyer H.U., Bernt E., Schmidt F., Stork H. (1974). D-Glucose: Determination with Hexokinase and Glucose-6-Phosphate Dehydrogenase.

[47] Redestig H., Kusano M., Fukushima A., Matsuda F., Saito K., Arita M. (2010). Consolidating metabolite identifiers to enable contextual and multi-platform metabolomics data analysis. BMC Bioinform..

[48] Ruperti B., Botton A., Populin F., Eccher G., Brilli M., Quaggiotti S., Trevisan S., Cainelli N., Guarracino P., Schievano E. (2019). Flooding Responses on Grapevine: A Physiological, Transcriptional, and Metabolic Perspective. Front. Plant Sci..

[49] Pedersen O., Rich S.M., Colmer T.D. (2009). Surviving floods: Leaf gas films improve O(2) and CO(2) exchange, root aeration, and growth of completely submerged rice. Plant J..

[50] Winkel A., Colmer T.D., Ismail A.M., Pedersen O. (2013). Internal aeration of paddy field rice (*Oryza sativa*) during complete submergence—Importance of light and floodwater O2. New Phytol..

[51] Kurokawa Y., Nagai K., Huan P.D., Shimazaki K., Qu H., Mori Y., Toda Y., Kuroha T., Hayashi N., Aiga S. (2018). Rice leaf hydrophobicity and gas films are conferred by a wax synthesis gene (LGF1) and contribute to flood tolerance. New Phytol..

[52] Mori Y., Kurokawa Y., Koike M., Malik A.I., Colmer T.D., Ashikari M., Pedersen O., Nagai K. (2019). Diel O2 Dynamics in Partially and Completely Submerged Deepwater Rice: Leaf Gas Films Enhance Internodal O2 Status, Influence Gene Expression and Accelerate Stem Elongation for ‘Snorkelling’ during Submergence. Plant Cell Physiol..

[53] Obata T., Fernie A.R. (2012). The use of metabolomics to dissect plant responses to abiotic stresses. Cell. Mol. Life Sci..

[54] Mackill D., Ismail A., Singh U., Labios R., Paris T. (2012). Development and Rapid Adoption of Submergence-Tolerant (Sub1) Rice Varieties. Adv. Agron..

[55] Van Dongen J.T., Frohlich A., Ramirez-Aguilar S.J., Schauer N., Fernie A.R., Erban A., Kopka J., Clark J., Langer A., Geigenberger P. (2009). Transcript and metabolite profiling of the adaptive response to mild decreases in oxygen concentration in the roots of arabidopsis plants. Ann. Bot..

[56] Rocha M., Licausi F., Araujo W.L., Nunes-Nesi A., Sodek L., Fernie A.R., van Dongen J.T. (2010). Glycolysis and the tricarboxylic acid cycle are linked by alanine aminotransferase during hypoxia induced by waterlogging of *Lotus japonicus*. Plant Physiol..

[57] Fukao T., Xu K., Ronald P.C., Bailey-Serres J. (2006). A variable cluster of ethylene response factor-like genes regulates metabolic and developmental acclimation responses to submergence in rice. Plant Cell.

[58] Alpuerto J.B., Hussain R.M.F., Fukao T. (2016). The key regulator of submergence tolerance, SUB1A, promotes photosynthetic and metabolic recovery from submergence damage in rice leaves. Plant Cell Environ..

[59] Kende H., van der Knaap E., Cho H.T. (1998). Deepwater rice: A model plant to study stem elongation. Plant Physiol..

[60] Bailey-Serres J., Voesenek L.A. (2010). Life in the balance: A signaling network controlling survival of flooding. Curr. Opin. Plant Biol..

[61] Woodward A.W., Bartel B. (2005). Auxin: Regulation, action, and interaction. Ann. Bot..

[62] Ludwig-Muller J. (2011). Auxin conjugates: Their role for plant development and in the evolution of land plants. J. Exp. Bot..

[63] Rampey R.A., LeClere S., Kowalczyk M., Ljung K., Sandberg G., Bartel B. (2004). A family of auxin-conjugate hydrolases that contributes to free indole-3-acetic acid levels during Arabidopsis germination. Plant Physiol..

[64] Staswick P.E. (2009). The tryptophan conjugates of jasmonic and indole-3-acetic acids are endogenous auxin inhibitors. Plant Physiol..

[65] Park J.E., Park J.Y., Kim Y.S., Staswick P.E., Jeon J., Yun J., Kim S.Y., Kim J., Lee Y.H., Park C.M. (2007). GH3-mediated auxin homeostasis links growth regulation with stress adaptation response in Arabidopsis. J. Biol. Chem..

[66] Ostrowski M., Ciarkowska A., Jakubowska A. (2016). The auxin conjugate indole-3-acetyl-aspartate affects responses to cadmium and salt stress in *Pisum sativum* L.. J. Plant Physiol..

[67] Kusano M., Fukushima A., Kobayashi M., Hayashi N., Jonsson P., Moritz T., Ebana K., Saito K. (2007). Application of a metabolomic method combining one-dimensional and two-dimensional gas chromatography-time-of-flight/mass spectrometry to metabolic phenotyping of natural variants in rice. J. Chromatogr. B Analyt. Technol. Biomed. Life Sci..

[68] Kusano M., Fukushima A., Arita M., Jonsson P., Moritz T., Kobayashi M., Hayashi N., Tohge T., Saito K. (2007). Unbiased characterization of genotype-dependent metabolic regulations by metabolomic approach in *Arabidopsis thaliana*. BMC Syst. Biol..

[69] Jonsson P., Johansson A.I., Gullberg J., Trygg J., A J., Grung B., Marklund S., Sjostrom M., Antti H., Moritz T. (2005). High-throughput data analysis for detecting and identifying differences between samples in GC/MS-based metabolomic analyses. Anal. Chem..

[70] Jonsson P., Johansson E.S., Wuolikainen A., Lindberg J., Schuppe-Koistinen I., Kusano M., Sjostrom M., Trygg J., Moritz T., Antti H. (2006). Predictive metabolite profiling applying hierarchical multivariate curve resolution to GC-MS data—A potential tool for multi-parametric diagnosis. J. Proteome Res..

[71] Redestig H., Fukushima A., Stenlund H., Moritz T., Arita M., Saito K., Kusano M. (2009). Compensation for systematic cross-contribution improves normalization of mass spectrometry based metabolomics data. Anal. Chem..

[72] Watanabe M., Kusano M., Oikawa A., Fukushima A., Noji M., Saito K. (2008). Physiological roles of the beta-substituted alanine synthase gene family in Arabidopsis. Plant Physiol..

[73] Kojima M., Kamada-Nobusada T., Komatsu H., Takei K., Kuroha T., Mizutani M., Ashikari M., Ueguchi-Tanaka M., Matsuoka M., Suzuki K. (2009). Highly sensitive and high-throughput analysis of plant hormones using MS-probe modification and liquid chromatography-tandem mass spectrometry: An application for hormone profiling in *Oryza sativa*. Plant Cell Physiol..

[74] Smyth G.K. (2004). Linear models and empirical bayes methods for assessing differential expression in microarray experiments. Stat. Appl. Genet. Mol. Biol..

[75] Smyth G.K., Gentleman R., Carey V., Dudoit S., Irizarry R., Huber W. (2005). Limma: Linear models for microarray data. Bioinformatics and Computational Biology Solutions using R and Bioconductor.

[76] Benjamini Y., Hochberg Y. (1995). Controlling the false discovery rate: A practical and powerful approach to multiple testing. J. R. Statist. Soc. B.

[77] Galili T., O’Callaghan A., Sidi J., Sievert C. (2018). heatmaply: An R package for creating interactive cluster heatmaps for online publishing. Bioinformatics.

[78] Johnson W.E., Li C., Rabinovic A. (2007). Adjusting batch effects in microarray expression data using empirical Bayes methods. Biostatistics.

