# Supplementary material for: Metabolite and Phytohormone Profiling Illustrates Metabolic Reprogramming as an Escape Strategy of Deepwater Rice during Partially Submerged Stress
Source: Metabolites. 2020 Feb 14;10(2):68. doi: 10.3390/metabo10020068 (PMC7074043; doi:10.3390/metabo10020068)
Supplement: Supplementary file 1 [file metabolites-10-00068-s001.zip › Fukushima_etal_SupplemtaryFilesPROOF/Fukushima_etal_SuppFigures_PROOF.pdf]

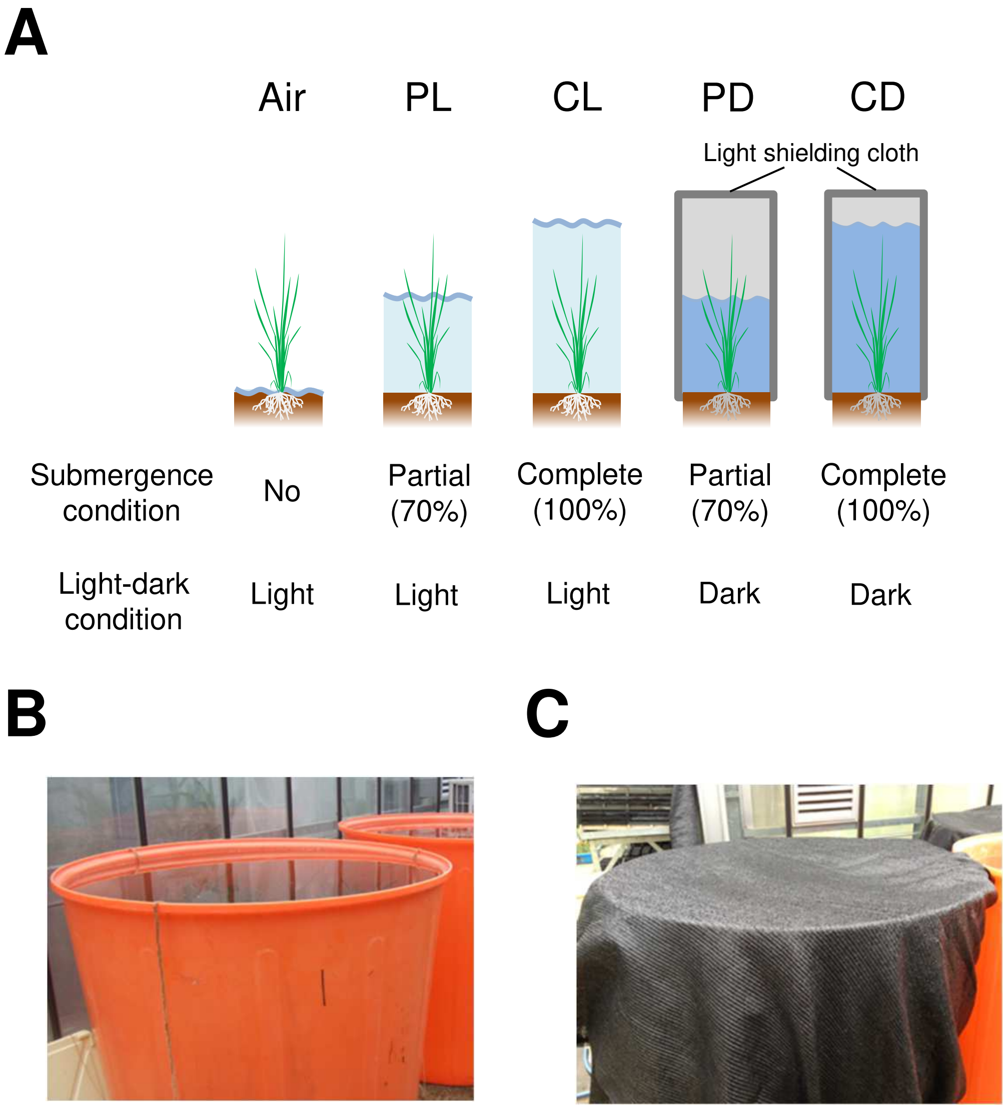

Supplementary Figure S1. Conditions of submergence treatments under light-dark conditions. (A) Schematic overview of each treatment. Percentages are based on ratio of shoot height to water level. Air, water level is under the soil surface; PL, PS treatment under light condition; CL, CS treatment under light condition; PD, PS treatment under dark condition; and CD, CS treatment under dark condition. (B) A tank for submergence treatment under light conditions. (C) A tank covered with light shielding cloth for submergence treatment under dark conditions.

A B

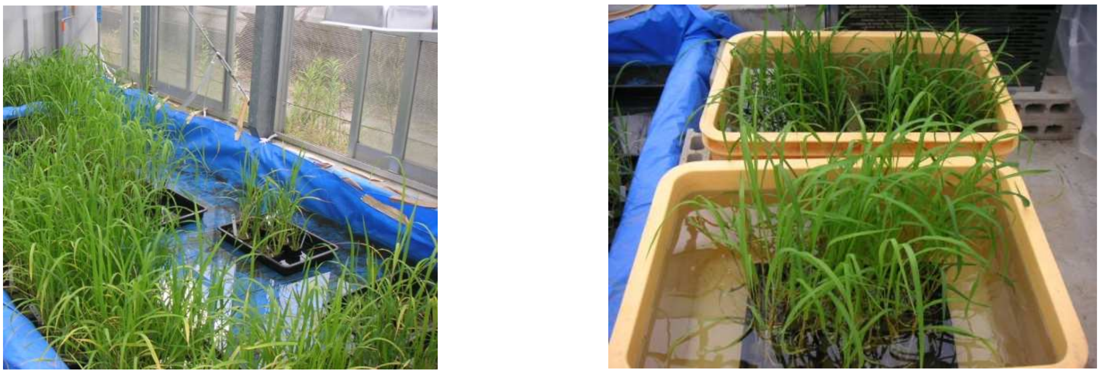

Supplementary Figure S2. Conditions of partial submergence treatments in the greenhouse. (A) Air condition. Water level is under the soil surface. (B) Partial submergence condition, 70% of aerial parts were soaked in water.

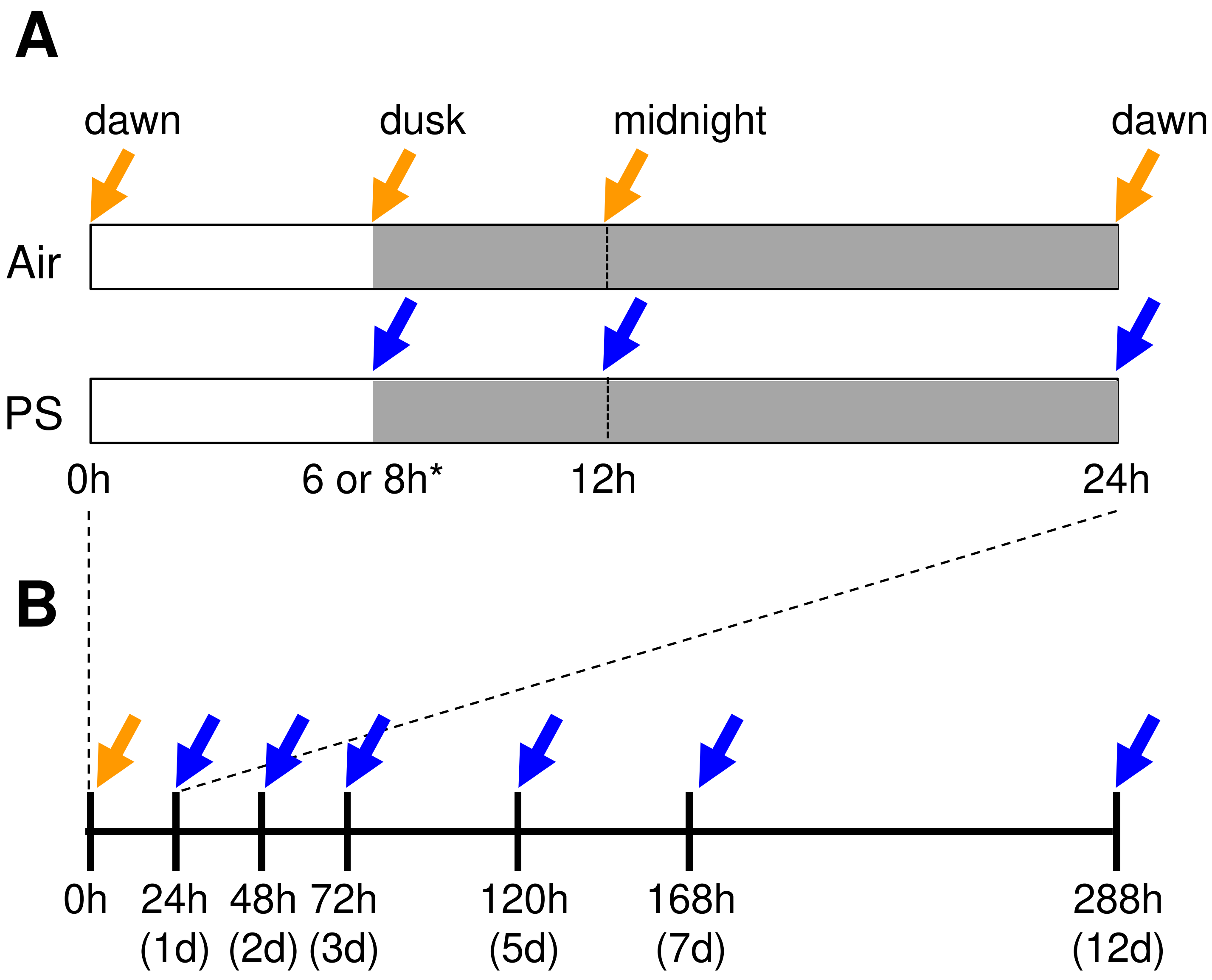

Supplementary Figure S3. Experimental design of short-term- (A) and long-term (B) experiments. The orange arrow represents sampling points under Air conditions, while the blue arrow represents sampling points under PS conditions. Abbreviations: Air, growth condition without submergence; PS, growth conditions by partial submergence treatment. *In the short-term experiment, internodes were sampled at 8 h after treatment.

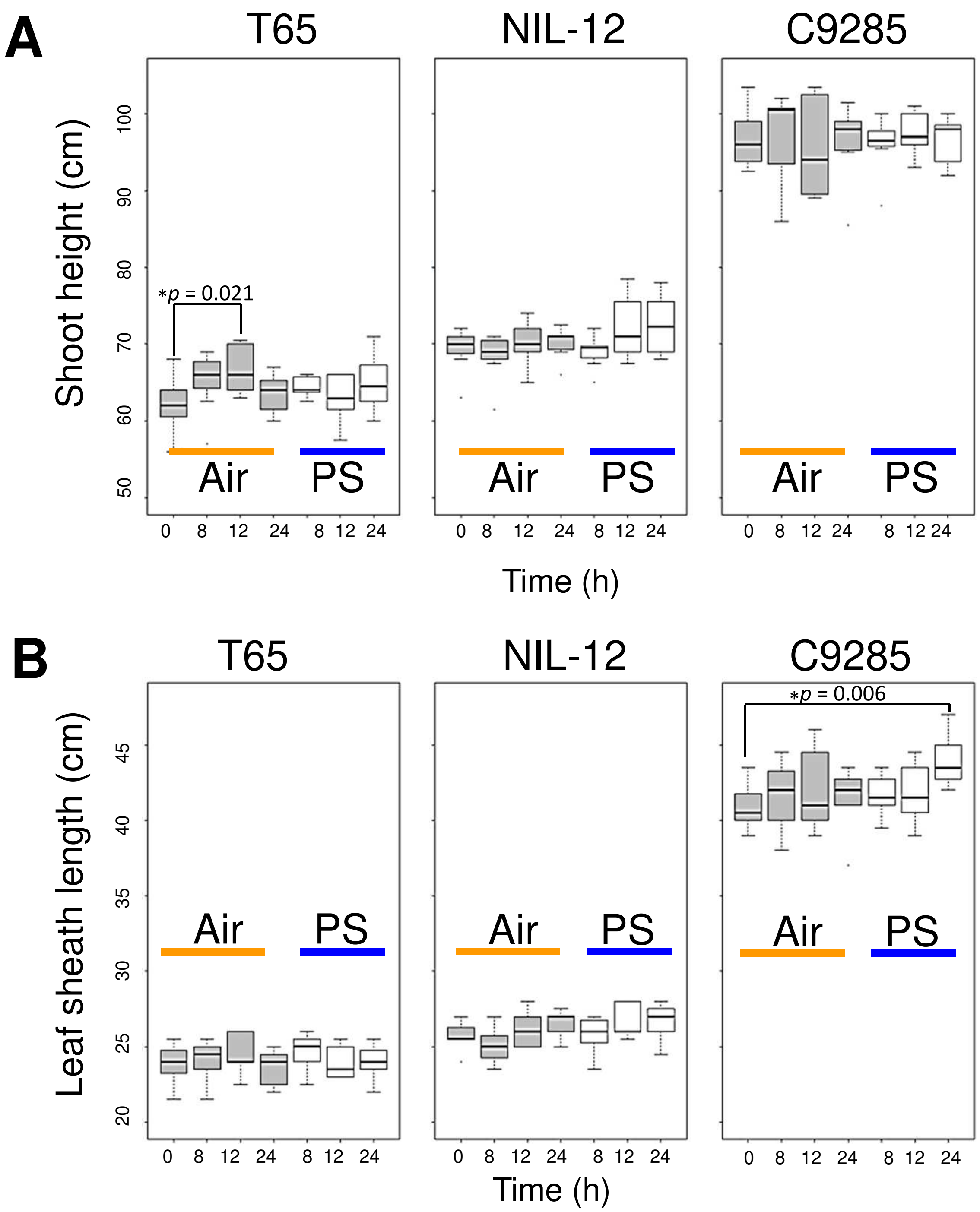

Supplementary Figure S4. Length of shoot and leaf sheath of T65, NIL-12, and C9285 in the short-term experiment. Shoot height (A) and LS length (B) were measured after Air or partial submergence treatments. Boxplots represent metabolite relative abundances. Asterisks show differences from the Air 0 h condition (Welch's *t*-test, $*p < 0.05$). Abbreviations: Air, growth condition without submergence; PS, growth conditions by partial submergence treatment. Biological replicates, $n = 6$.

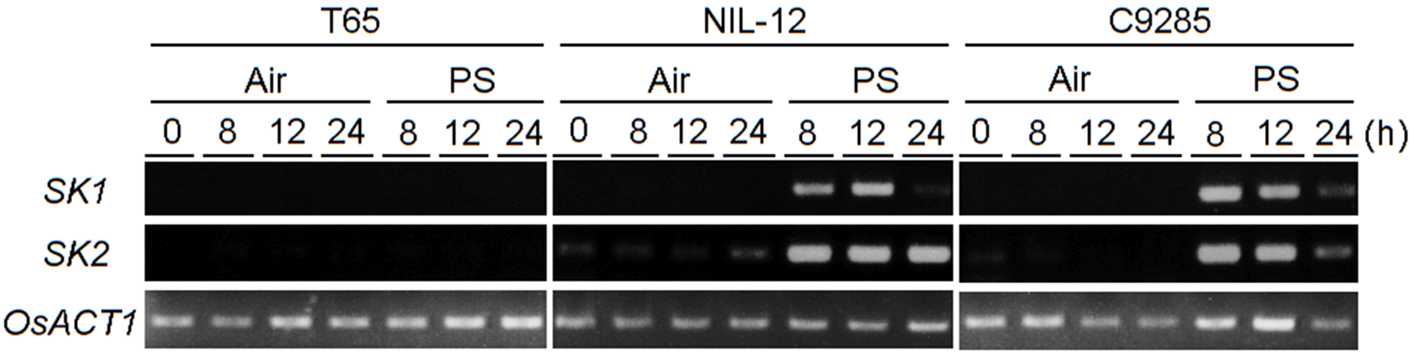

Supplementary Figure S5. Semi-quantitative RT-PCR analysis of *SK1*/2 in T65, NIL-12, and C9285. T65, NIL-12, and C9285, plants at the 8-leaf stage were subjected to each condition. Semi-quantitative RT-PCR was performed with 35, 35, and 25 cycles for *SK1*, *SK2*, and *OsACT1*, respectively. *OsACT1* (rice Actin1 gene) was used for an internal standard control. Air, growth condition without submergence; PS, growth conditions by partial submergence treatment.

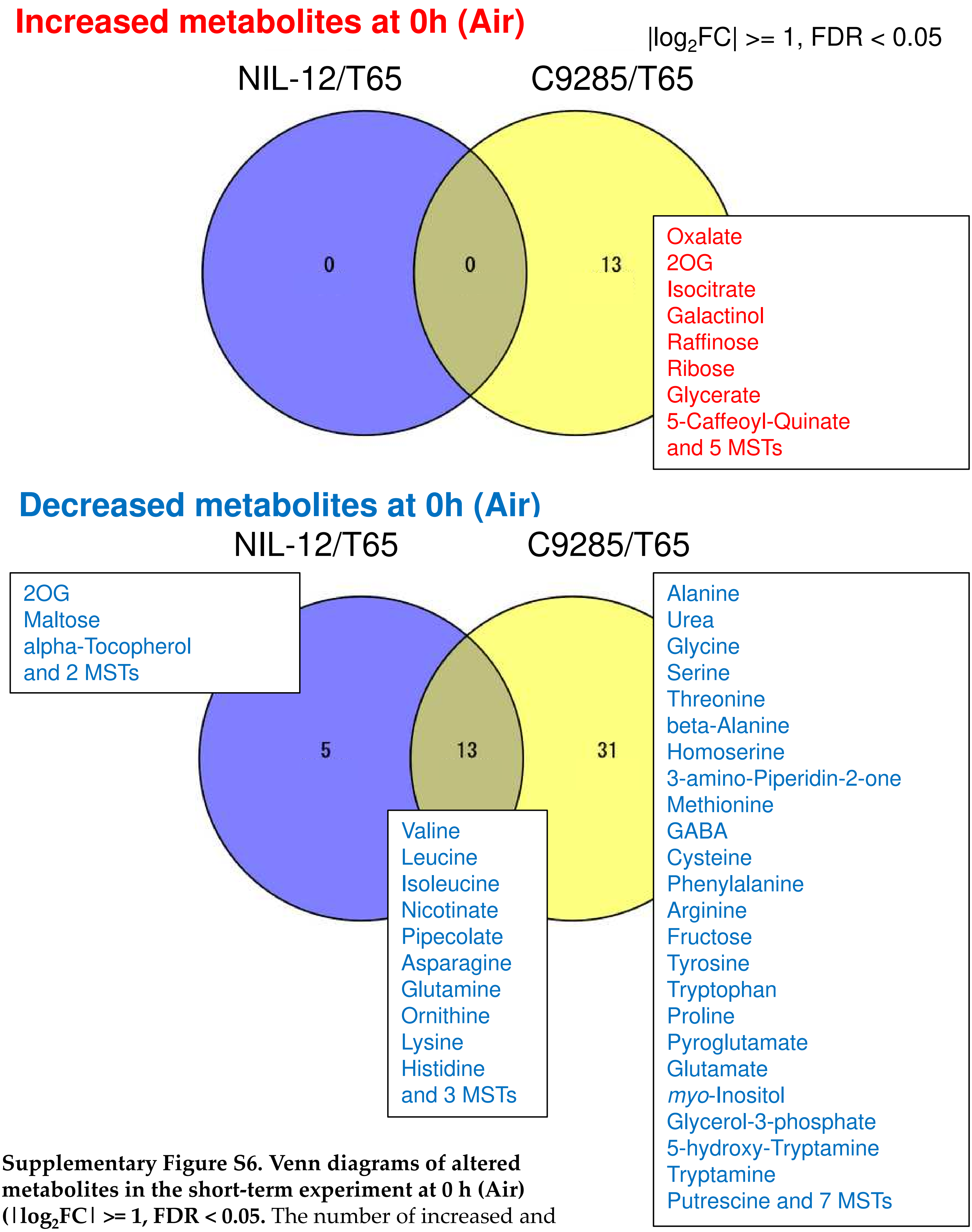

Supplementary Figure S6. Venn diagrams of altered metabolites in the short-term experiment at 0 h (Air) (|$\log_2$FC| >= 1, FDR < 0.05. The number of increased and decreased metabolites is shown. FC, fold-change; FDR, false discovery rate; 2OG, 2-oxoglutarate; GABA, γ-aminobutyric acid; MST, mass spectral tag.

Increased metabolites at 8h (PS)

|log$_2$FC| >= 1, FDR < 0.05

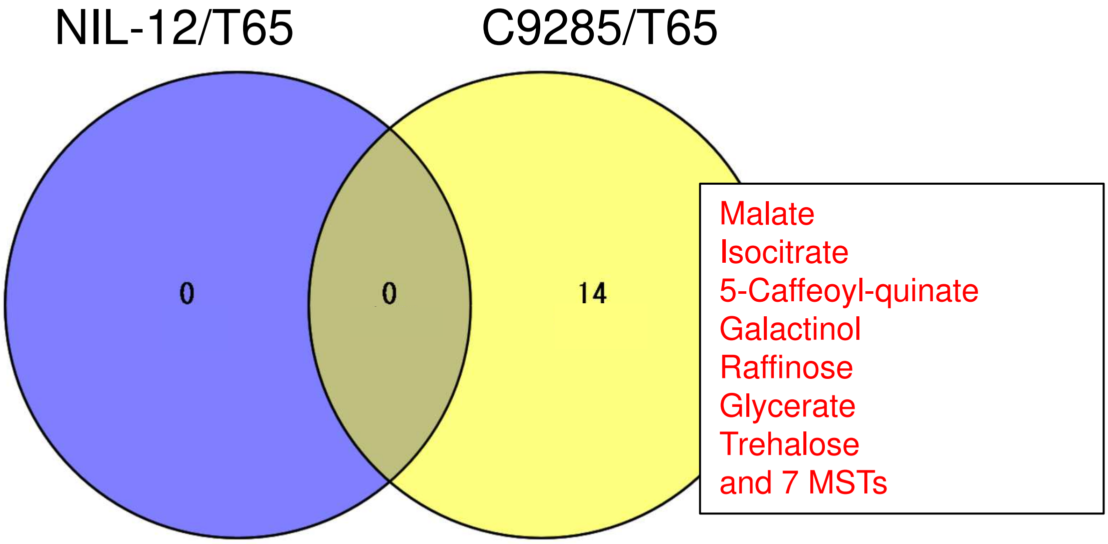

Decreased metabolites at 8h (PS)

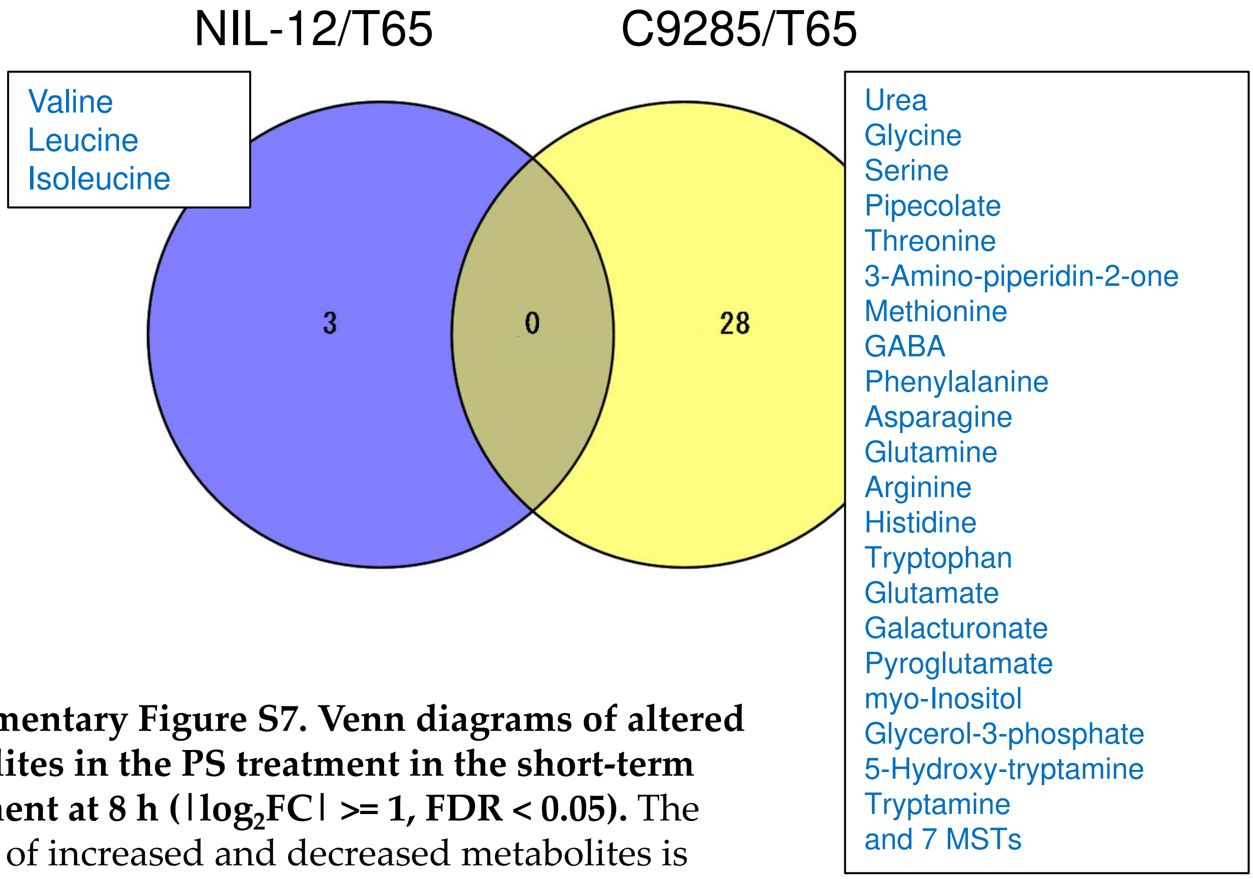

Supplementary Figure S7. Venn diagrams of altered metabolites in the PS treatment in the short-term experiment at 8 h (|log$_2$FC| >= 1, FDR < 0.05). The number of increased and decreased metabolites is shown. FC, fold-change; FDR, false discovery rate; PS, partial submergence; GABA, γ-aminobutyric acid; MST, mass spectral tag.

Increased metabolites at 12h (PS)

$|\log_2 FC| >= 1$, FDR < 0.05

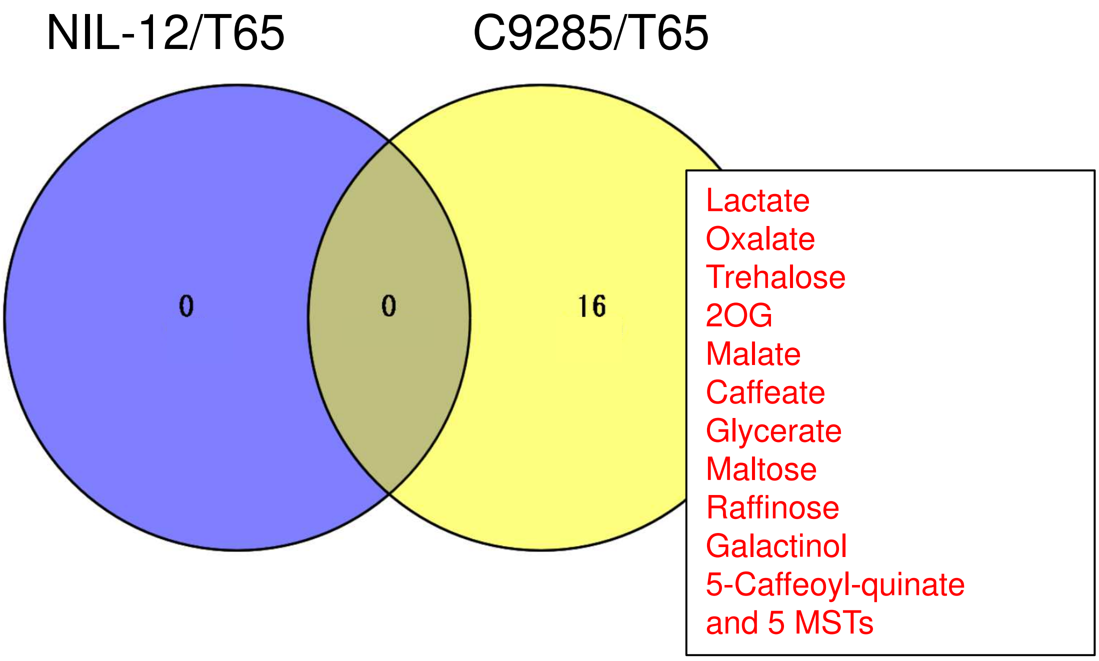

Decreased metabolites at 12h (PS)

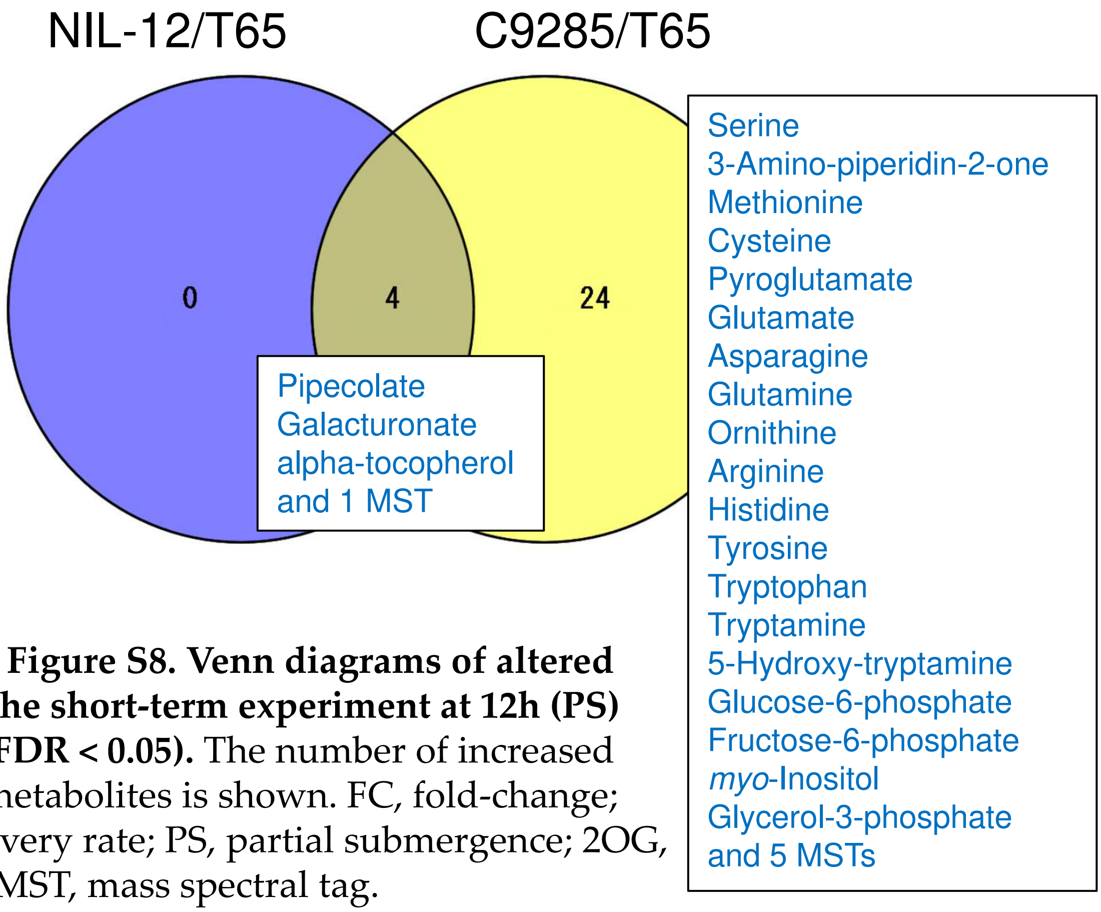

Supplementary Figure S8. Venn diagrams of altered metabolites in the short-term experiment at 12h (PS) ($|\log_2 FC| >= 1$, FDR < 0.05). The number of increased and decreased metabolites is shown. FC, fold-change; FDR, false discovery rate; PS, partial submergence; 2OG, 2-oxoglutarate; MST, mass spectral tag.

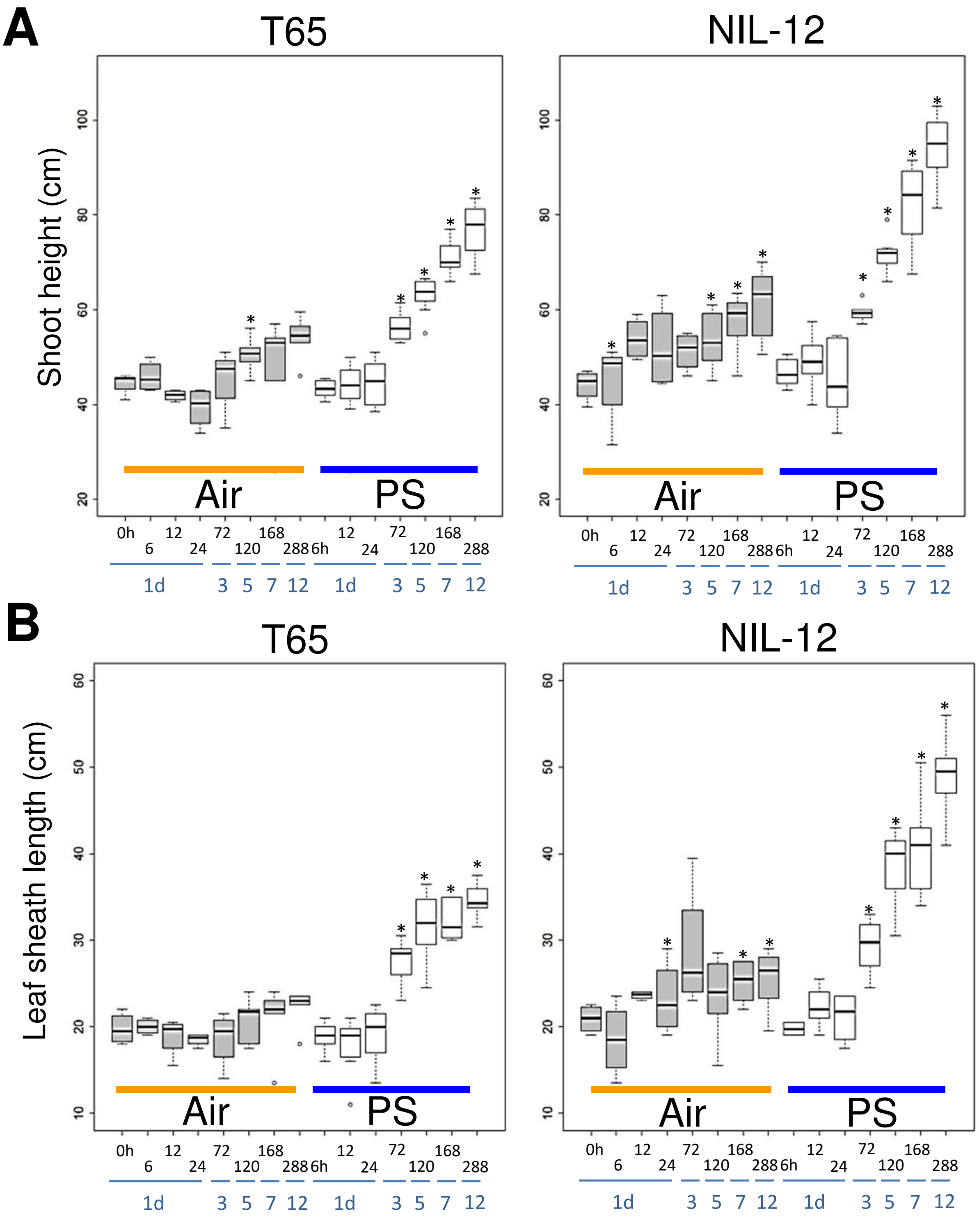

Supplementary Figure S9. Length of shoot and leaf sheath (LS) of NIL-12 and T65 in the long-term experiment. Shoot height (A) and LS length (B) were measured after air or partial submergence treatments. Boxplots represent shoot and LS length. Asterisks represent statistically significant differences from the Air 0 h condition (Welch's *t*-test, $^*p < 0.05$). Air, air conditions; PS, growth conditions by partial submergence treatment. Biological replicates, n = 4-8.

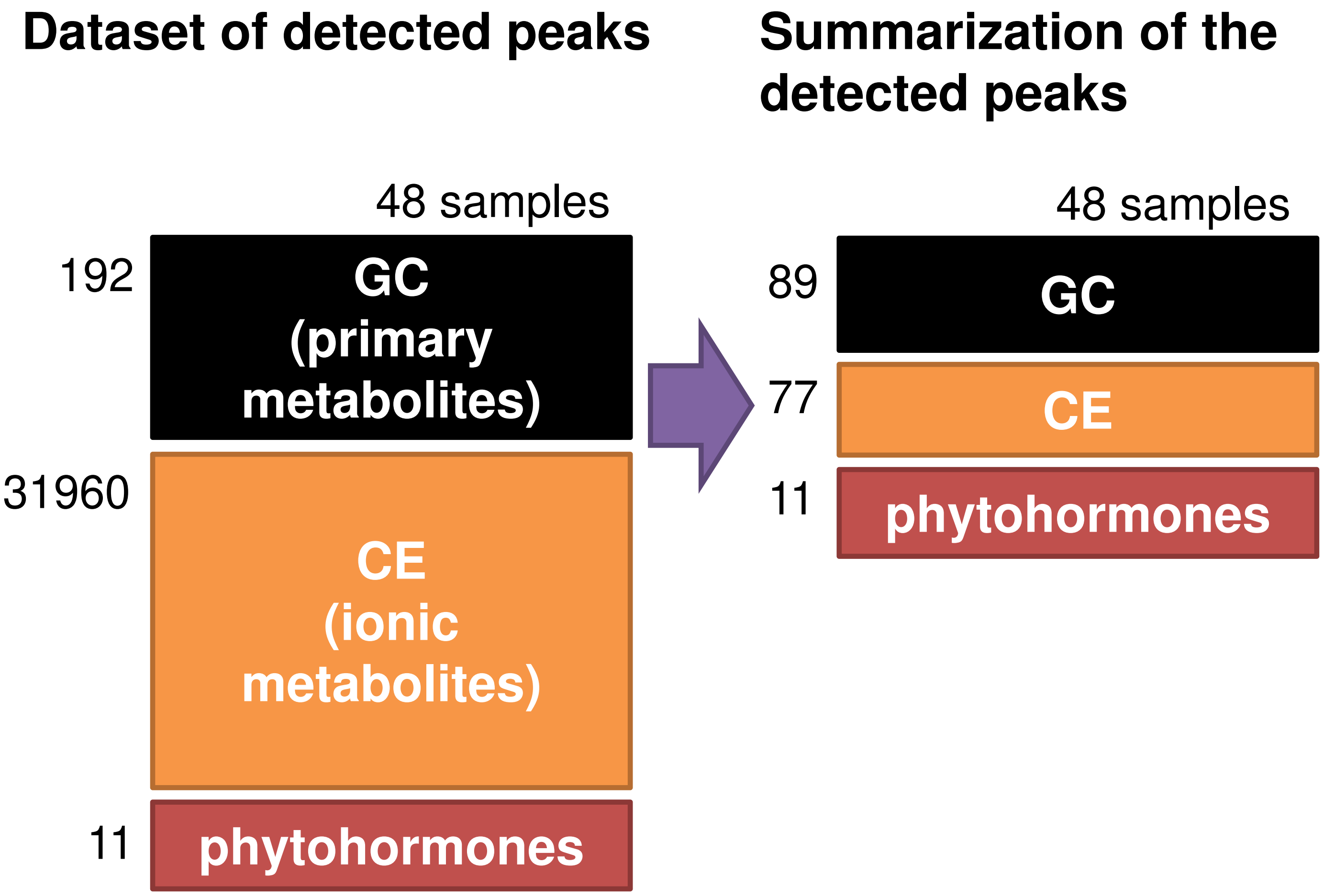

Supplementary Figure S10. Data summarization of the metabolite and phytohormone dataset. We used metabolite and phytohormone profiles after summarizing with the MetMask tool [46] for statistical analysis. Abbreviations: GC, GC-MS analysis; and CE, CE-MS analysis.

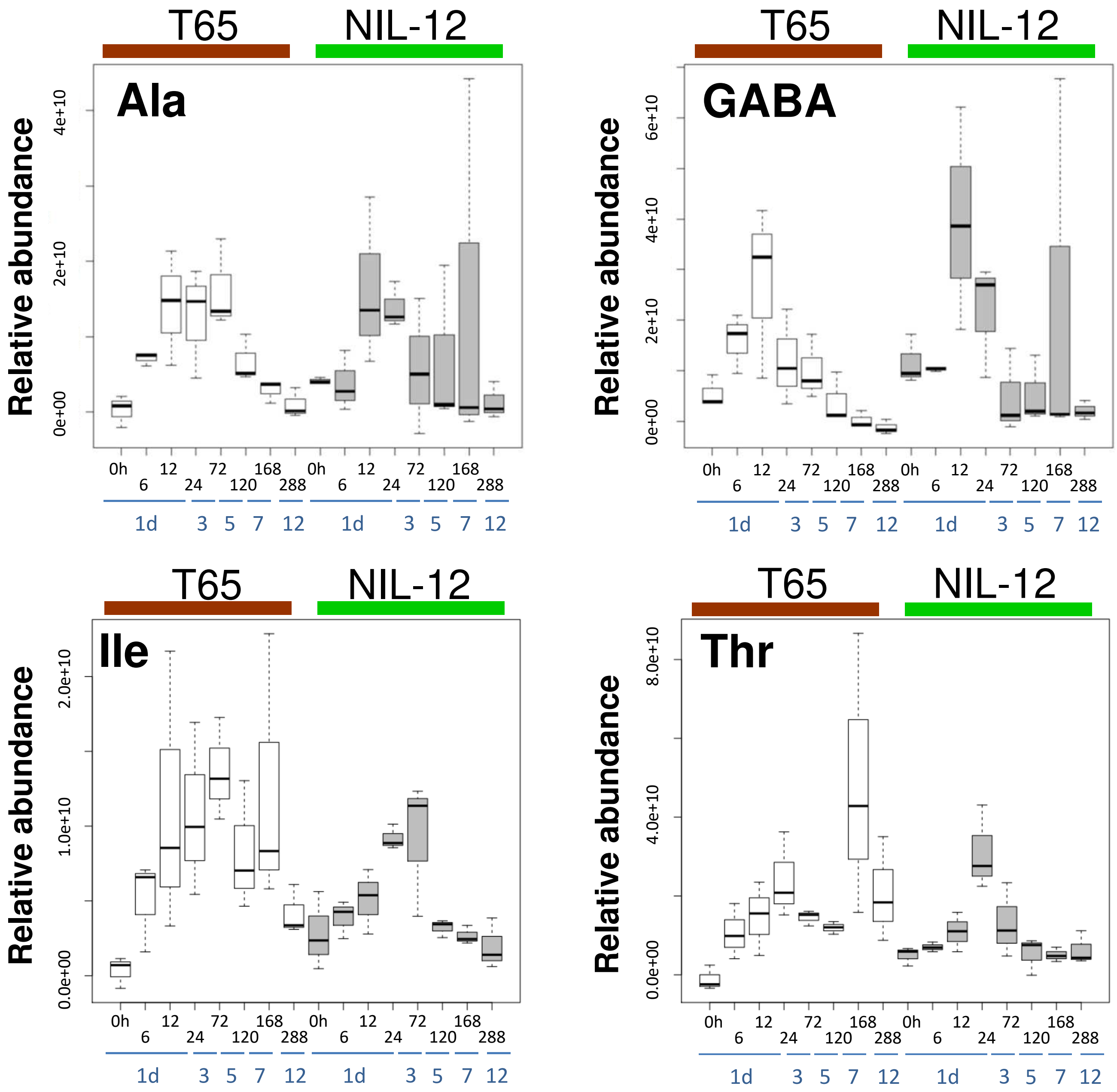

Supplementary Figure S11. Changes of known hypoxia-inducible metabolites described in Barding, et al (2012) [38] and Ruperti, et al (2019) [48]. Boxplots represent the metabolite relative abundances. Air, growth condition without submergence; PS, growth conditions by partial submergence treatment; Ala, Alanine; GABA, γ-aminobutyric acid; Ile, isoleucine; Thr, threonine; Val, valine; Gln, glutamine; Glu, glutamate. Biological replicates, *n* = 3.

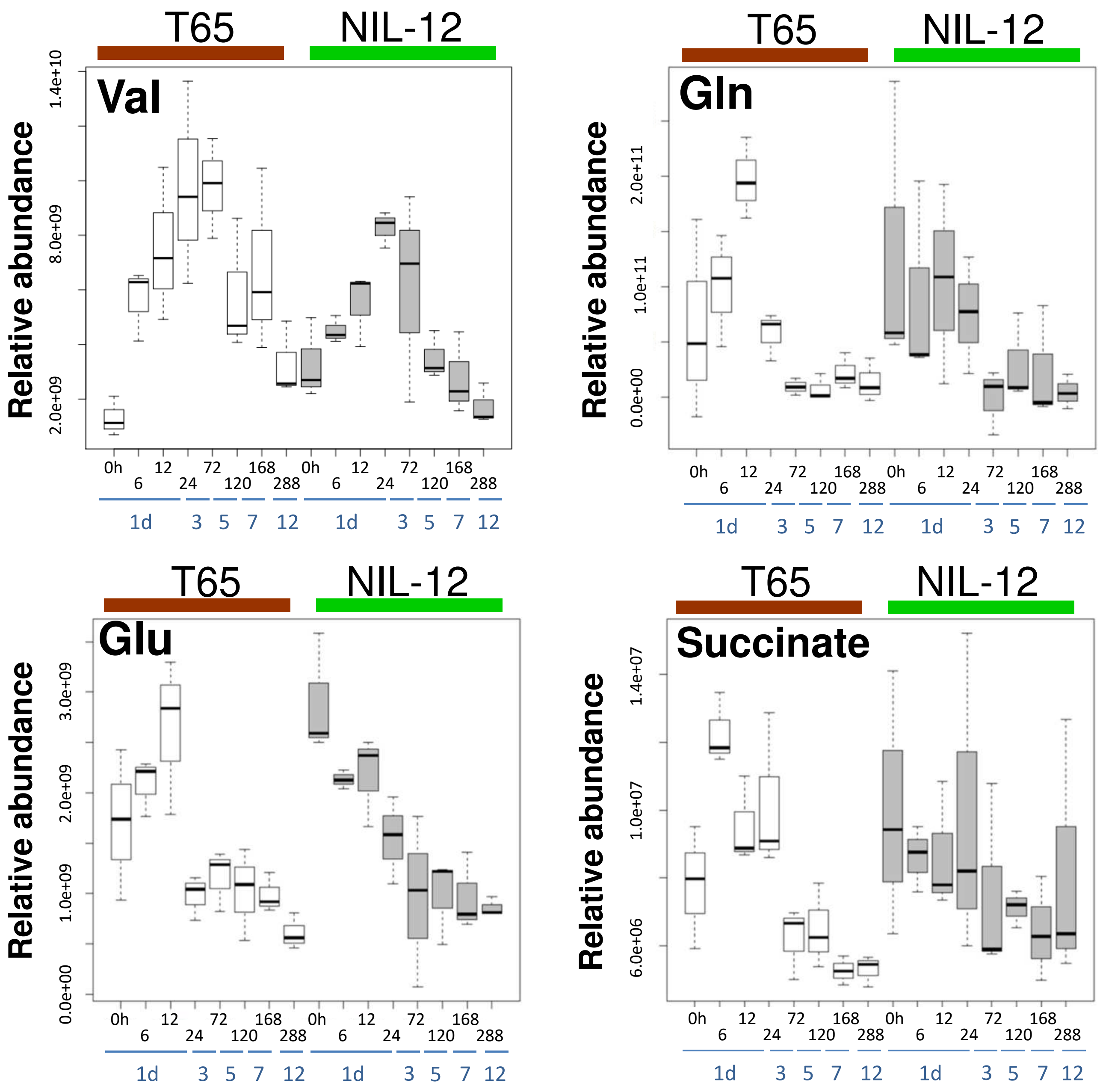

Supplementary Figure S11. (continued).
